# Virtual Care Initiatives for Older Adults in Australia: Scoping Review

**DOI:** 10.2196/38081

**Published:** 2023-01-18

**Authors:** Feby Savira, Adyya Gupta, Cecily Gilbert, Catherine E Huggins, Colette Browning, Wendy Chapman, Terry Haines, Anna Peeters

**Affiliations:** 1 Deakin Health Economics Institute for Health Transformation Deakin University Burwood Australia; 2 Global Centre for Preventive Health and Nutrition Institute for Health Transformation Deakin University Geelong Australia; 3 Centre for Digital Transformation of Health University of Melbourne Melbourne Australia; 4 Health Innovation and Transformation Centre Federation University Ballarat Australia; 5 Institute of Health and Wellbeing Federation University Ballarat Australia; 6 National Centre for Healthy Ageing Monash University Frankston Australia; 7 School of Primary and Allied Health Care Monash University Frankston Australia; 8 Institute for Health Transformation Deakin University Geelong Australia

**Keywords:** virtual care, older adults, Australia, mobile phone

## Abstract

**Background:**

There has been a rapid shift toward the adoption of virtual health care services in Australia. It is unknown how widely virtual care has been implemented or evaluated for the care of older adults in Australia.

**Objective:**

We aimed to review the literature evaluating virtual care initiatives for older adults across a wide range of health conditions and modalities and identify key challenges and opportunities for wider adoption at both patient and system levels in Australia.

**Methods:**

A scoping review of the literature was conducted. We searched MEDLINE, Embase, PsycINFO, CINAHL, AgeLine, and gray literature (January 1, 2011, to March 8, 2021) to identify virtual care initiatives for older Australians (aged ≥65 years). The results were reported according to the World Health Organization’s digital health evaluation framework.

**Results:**

Among the 6296 documents in the search results, we identified 94 that reported 80 unique virtual care initiatives. Most (69/80, 89%) were at the pilot stage and targeted community-dwelling older adults (64/79, 81%) with chronic diseases (52/80, 65%). The modes of delivery included videoconference, telephone, apps, device or monitoring systems, and web-based technologies. Most initiatives showed either similar or better health and behavioral outcomes compared with in-person care. The key barriers for wider adoption were physical, cognitive, or sensory impairment in older adults and staffing issues, legislative issues, and a lack of motivation among providers.

**Conclusions:**

Virtual care is a viable model of care to address a wide range of health conditions among older adults in Australia. More embedded and integrative evaluations are needed to ensure that virtually enabled care can be used more widely by older Australians and health care providers.

## Introduction

Australia has one of the most complex and decentralized health care systems among the Organisation for Economic Co-operation and Development countries [1,2]. Care decentralization may result in duplication efforts, inefficiency, or poor coordination in service delivery, especially for vulnerable populations and those in rural or remote areas [2]. Virtual care offers a potential solution for streamlining care processes and improving access to care [3]. It is broadly defined as the remote provision of care assisted by information technology [4]. Australia has undergone a rapid shift toward the adoption of virtual health care services in the last 10 years [5]. Strategies to improve uptake of virtual care among older adults may be particularly beneficial, as they are major consumers of health care resources in Australia (30% of unreferred general practitioners and 46% of specialist services in 2019-2020), and are known to experience poor coordination of care [6,7]. Over one-third of older Australians reside in rural or remote areas [8], further compounding problems with access to care.

It is unclear how widely virtual care has been implemented or evaluated in the care of older Australians. There is also a lack of clarity regarding the most appropriate type of virtual care to address the complex health care needs of older people, particularly owing to disability, frailty, long-term health conditions, cognitive decline [9], and higher likelihood of a “digital divide” [10]. Therefore, in this scoping review, we aimed to provide a brief appraisal of virtual care initiatives for older adults in Australia across a wide range of health conditions and modalities and identify key challenges and opportunities for wider adoption at both patient and system levels.

## Methods

### Search Strategy

A scoping review methodology was chosen for this review to capture a wide range of virtual care initiatives for the complex care needs of older people in Australia [11]. Five databases (MEDLINE, Embase, PsycINFO, CINAHL, and AgeLine) and gray literature were screened from January 1, 2011, to March 8, 2021, to identify studies evaluating virtual care initiatives for older adults in Australia. The search strategy was built using a combination of subject headings and keywords of the 4 concepts of “virtual care,” “initiatives,” “older adults,” and “Australia” (see Table S1 in Multimedia Appendix 1 for the full search strategy). For gray literature, we used Google Advanced Search and searched key Australian governmental, educational, and organizational domains. We also searched The Analysis & Policy Observatory, Informit, and International HTA Database.

### Study Selection

The virtual care initiatives included were limited to those relevant for older adults in Australia. Therefore, any Australian study that exclusively included participants aged ≥65 years or had participants with a mean or median age of ≥65 years or other clearly stated definition of older adults (eg, aged ≥55 years) were eligible for inclusion. Virtual care included any form of technology-mediated care modality including videoconference, telephone or smartphone, device use (including remote monitoring), and other eHealth interventions (eg, apps or websites). Delivery of care included symptom or progress monitoring, education, support, and disease management and treatment. Studies were included if the outcomes were obtained from an older adult’s perspective. The inclusion and exclusion criteria are presented in Table S2 in Multimedia Appendix 1.

The PRISMA-ScR (Preferred Reporting Items for Systematic Reviews and Meta-Analyses extension for Scoping Reviews) checklist guided the reporting of this scoping review [12]. Search results were imported into Covidence, and duplicates were removed (see Figure S1 in Multimedia Appendix 1 for PRISMA flowchart). Using a screening checklist, 2 reviewers independently screened the titles and abstracts to identify studies for inclusion. Any discrepancies were resolved through discussion, with consultation from a third reviewer, where needed. The search and screening process was cross-checked by a health information specialist.

### Data Extraction

Two reviewers extracted study details from the selected reports using a standardized extraction form. To streamline the scoping review process, a follow-up to study authors was not conducted for missing or incomplete data or information. The following information was extracted: publication details, population characteristics, virtual care details, setting, key findings, and cost assessment (if any). The other key measures extracted were acceptability (for patients or providers), adoption or scalability, and funding source.

### Data Analysis

The results were narratively synthesized and presented according to the World Health Organization (WHO) digital health evaluation framework [13]. The WHO framework provides a thorough evaluation guideline of the outputs and impacts of digital health interventions across various domains, including user satisfaction, process improvements, health outcomes, and cost-effectiveness (see Table S3 in Multimedia Appendix 1 for full definitions of each item) [13]. The framework items included in this study were intervention delivery, content, cost assessment, user feedback, and limitation for delivery at scale. A brief synthesis of other evaluation items is provided in Multimedia Appendix 1. The effectiveness of virtual care initiatives was also summarized in terms of health, behavioral, or any health service use outcomes (see Table S2 in Multimedia Appendix 1 for definitions). To note, virtual care initiatives that were delivered as a stand-alone intervention was considered similar or comparable (ie, noninferior) to in-person care if similar outcomes were yielded. The rationale is similar to the measurement of effects in noninferiority trials, wherein if a stand-alone virtual care intervention can provide marginal benefits akin to delivering in-person care alone, then it should be considered at least as effective as in-person (standard) care [14]. However, interventions involving the addition of virtual care to in-person care, compared with in-person care alone, were evaluated for superiority rather than noninferiority [14]. If the combination of virtual care and in-person care does not yield any additional health or behavioral outcome improvements compared with in-person care alone, it was considered inferior, as the intervention (as a whole) cannot compensate for the required extra time, effort, or potential costs to deliver the added virtual care initiative without any additional health benefit and would be no better than delivering a standard care intervention alone.

The initiatives were also grouped according to the following categories:

- *Communication technologies*: initiatives that enable remote interactions between patient and health care provider.
- *Information and data sharing*: initiatives that facilitate remote sharing of patients’ medical and health care information across care providers or relevant stakeholders.
- *Remote monitoring*: initiatives that use hardware and software to allow remote measurement or documentation of a patient’s physiology. The information is either transmitted in real time or stored for subsequent transmission.

We also determined whether the intervention delivery was synchronous (ie, where patient-provider interactions occur in real time), asynchronous (ie, not in real time), or a combination of both. In scoping reviews, assessing the risk of bias in studies is not mandatory, and no risk of bias assessment was conducted for this topic [12].

## Results

### Overview

Out of 6296 documents, 94 references met the inclusion criteria. We identified 80 unique Australian virtual care initiatives for older adults, of which 9 (11%) initiatives were considered mature (well-embedded or a widely used government-initiated intervention), and others (n=71, 89%) were pilot evaluations (small-scale or feasibility studies). Table 1 provides a summary of the identified initiatives and the key findings of the studies.

**Table 1:** Summary of virtual care initiatives in Australia (n=80).

Reference, study design, or setting		Older adult sample^a^	Virtual care initiative^b^	Purpose of virtual care	Key findings (including cost assessment, if any)
**Videoconference**					
	Banbury et al [15], 2017 Community Mixed methods, nested within a nonrandomized trial	Mean age of 73 years, community dwelling with long-term conditions 52 patients	Videoconference Communication technologies, synchronous, pilot N/A^c^	Increase people’s social networks, which contribute to their social support for health and their engagement and perception of these networks	Unchanged—only a difference of 2.0 network members (SD 3.9), range −2 to 12. Ranking of top 3 social network, (ie, health professionals, close family, and partners remained the same). Participants identified friends and wider family as more important to manage their chronic condition postintervention. Higher costs—the true cost of delivery was US $75/week or US $11/day per participant (US $58/week or US $8/day, excluding overheads). Cost may be justifiable for early discharge or hospital avoidance programs for patients at high risk of hospitalization. The weekly cost of providing videoconferencing services only is US $36, including overheads.
	Dham et al [16], 2018 Community or in-hospital or clinic, mixed, retrospective analysis of usage pattern and qualitative survey or feedback of consultation	Mean age of 76 years, needing psychiatric care 134 consults of 101 patients	Videoconference Communication technologies, synchronous, pilot N/A	Improve psychiatric care delivery from the perspective of a community-based program (not in nursing homes or inpatients)	Intervention feedback—mean scores ranged from 3. 9-4.4 for patients, 4.4-4.7 for clinicians, and 3.7-4.5 out of 5 for psychiatrists. Feedback from inpatients was significantly lower than that from outpatients, and they were significantly less satisfied with the wait time and visual clarity.
	Moyle et al [17], 2020 RACF^d^ semistructured interviews and thematic analysis	Mean age of 89 years, living in long-term care facilities 6 patients	Videoconference Communication technologies, synchronous, pilot N/A	Assess viability of using videoconference in long-term care population	Intervention feedback—videoconference is regarded positively and seen as a good way of communicating with family or friends. Use of tablets is inhibited by age-related cognitive decline and physical frailty. It may be an unfamiliar technology for many older residents, and practice and staff assistance are required. There were general concerns about privacy and cyber security.
	Martin-Khan et al [18], 2012 In-hospital or clinic Prospective cohort	Mean age of 76 years, potentially with dementia 205 total—100 intervention and 105 control	Videoconference Communication technologies, synchronous pilot Face-to-face consultation	Validate the diagnosis of dementia via video call using interrater agreement	Noninferior—acceptable agreement between videoconference and comparator group for diagnosis of dementia. The summary Cohen κ statistic was 0.51.
	Venuthurupalli et al [19], 2018 In-hospital or clinic Observational registry–based study	Mean age of 65 years, has CKD^e^ 1051 total—234 intervention and 817 control	Videoconference Communication technologies, synchronous, pilot Face-to-face consultation	Improve access to nephrology care	Improved—reduced mean number of admissions per patient in intervention group (1.63 vs 2.25 in comparator), but longer mean length of stay (5.5 vs 4.0 days). After 5.9 years follow-up, there were lower rates of renal replacement therapy in intervention (2.0 vs 3.5 cases per 100 patient-years), as well as mortality (4.5 vs 5.3 cases per 100 patient-years) and dialysis initiation (5.1% vs 9.9%). Lower costs—direct costs saved in the form of fuel subsidy, accommodation, and travel arrangements.
	Wundersitz et al [20], 2020 Community Feasibility study based on the Bowen framework	Age range of 52-75 years, under physiotherapy, dietetics, or speech pathology care 10 patients; 5 allied health clinicians	Videoconference Communication technologies, synchronous, pilot N/A	Facilitate rehabilitation for older adults via remote means	Intervention feedback—telerehabilitation is equivalent to face-to-face home visit to replace a session providing education, advice, or talking-based therapy and if the client does not have communication impairment. Reasons for declining telerehabilitaiton included anxiety and lack of confidence in using technology, not being interested, wanting hands-on therapy, and not having a private place at home. Limitations include technical problems (primarily owing to poor internet speed), limited ability to incorporate objects into session and difficulty positioning camera to see different parts of house or client’s body.
	Bladin et al [21] (pilot), 2015, and (implementation) [22], 2020 In-hospital or clinic Historical controlled cohort, comparison of 12-month control period vs initial 12 months of full implementation	Age range of 63-86 years, needing acute stroke care 6065 total—3178 in intervention period, 2887 in control period	Videoconference Communication technologies, information and data sharing, synchronous, mature N/A (Bladin et al [21]); face-to-face consultation (Nagao et al [23])	Enhance acute stroke care in regional Australia	Improved—larger proportion received thrombolysis during intervention than control period (37% vs 30%), with smaller proportions with symptomatic intracerebral hemorrhage (4% vs 16%) or died in hospital (6% vs 20%). Door-to-computed tomography scan time and door-to-needle time for stroke thrombolysis were also shorter during the intervention.
	Nagao et al [23] (rural), 2012 (same initiative as Bladin et al [21]) Medical file audit	Median age of 77 years, needing acute stroke care 275 total—130 in intervention period and 145 in control period	Videoconference Communication technologies, information and data sharing, synchronous, mature N/A (Bladin et al [21]); face-to-face consultation (Nagao et al [23])	Provide remote access to neurologists in rural areas	Noninferior—in intervention group, 24 patients had telestroke activated and 8 underwent thrombolysis vs no thrombolysis in control. No hemorrhages or deaths reported in both groups. Median door-to-computed tomography time did not differ between groups. Higher costs—total set-up cost was US $4894, and recurring cost was US $1341 per annum, excluding costs of preexisting computers, laptops, or reimbursement of neurologists.
	Burns et al [24], 2012 (pilot), and (cost analysis and trial) [25,26], 2017 In-hospital or clinic Randomized controlled trial	Mean age of 65 years, patients with head and neck cancer under speech pathology care 82 total—43 intervention and 39 control	Videoconference Communication technologies, information and data sharing, synchronous, mature Face-to-face consultation	Support the swallowing and communication management of patients with head and neck cancer	Noninferior—an equivalent positive increase in quality of life (0.04) was reported for both groups. Lower costs—an average cost saving of US $67 per referral using videoconference. Telepractice provided savings of US $40 in speech pathology service costs per referral, US $11in travel costs, and US $16 in time or wages for the patient.
	Host et al [27], 2018 In-hospital or clinic questionnaire	Mean age of 65 years, needing opthalmology care 109 (80% survey participation rate)	Videoconference Communication technologies, synchronous, pilot N/A	Provide specialist eye services outside of major cities	Intervention feedback—69% participants were “very satisfied” and 25% “satisfied.” Familiarity and support provided by providers were comforting. No demographic or follow-up variables were predictive of greater total satisfaction. However, participants who were older felt they could easily explain their medical problems to the doctor in the video consultation and believed that telemedicine enabled them to save money and time; they were also more likely to report higher overall satisfaction.
	Jiang et al [28], 2020 Community Prospective mixed methods pilot study	Mean age of 70.4 years with average survival of 5.8 months, needing palliative care 21 patients completed the study; 14 intervention and 7 standard care	Videoconference Communication technologies, synchronous, pilot Face-to-face consultation	Improve access to palliative care	Improved—less functional decline at 2 weeks and 3 months after the intervention vs comparator. At 30 days before death, functional status remained better in the intervention group, with fewer per capita community palliative care nursing visits (5.46 vs 9.32), general practitioner visits (0.13 vs 3.88), and hospital admissions (0.02 vs 0.2).
	Lillicrap et al [29], 2020 In-hospital or clinic Analysis of before and after data	Mean age of 70 years, needing acute stroke care 539 with complete data	Videoconference, 24/7 stroke triage in rural hospitals Communication technologies, information and data sharing, synchronous, mature N/A	Reduce door-to-treatment times and poorer patient outcomes in after-hours admission of stroke	Noninferior (no comparator)—no difference between in-hours, out-of-hours, and proportions of patients confirmed to have strokes or selected for reperfusion therapies at 3 months.
	Mariño et al [30] (pilot), 2014, and (cost analysis) [31], 2016 RACF Feasibility and cost analysis	RACF residents, needing dentistry care 100 for cost analysis, 50 for feasibility study	Videoconference Communication technologies, information and data sharing, synchronous, pilot Face-to-face consultation	Alternative way for virtual oral examination to develop a treatment plan	Intervention feedback—teledentistry is a feasible and reliable alternative. Most residents were highly satisfied, but 3 were dissatisfied (reason was because of a lack of immediate feedback on the examination). Most agree it was easy to understand remote communications and enjoyed the convenience. Most sessions needed an oral health professional to manipulate the intraoral camera despite provision of training and written instructions. Mixed—net cost for teledental asynchronous patient from a health care perspective was estimated to be Aus $32.4 (US $22) (vs Aus $36.6 [US $25] if face-to-face). Total cost of real-time remote oral examination would be Aus $41.3 (US $28) per resident. Staff time costs accounted for 80% of the total intervention delivery cost in both virtual and in-person options.
	Sabesan et al [32], 2012 In-hospital or clinic Descriptive analysis	Median age of 67 years, patients with cancer living in rural towns 158 patients	Videoconference Communication technologies, synchronous, pilot N/A	Improve access to rural cancer care	Improved (no comparator)—10 patients were consulted urgently, and treatment plans initiated locally, thus avoiding interhospital transfers. All were seen within 24 hours.
	Stillerova et al [33], 2016 Community Descriptive analysis and interview	Median age of 69 years, with Parkinson disease 11 patients	Videoconference Communication technologies, synchronous, pilot Face-to-face consultation	Provide timely and efficient monitoring and support for people with Parkinson disease	Noninferior—all Montreal Cognitive Assessment items (for Parkinson disease) could be completed over videoconference, with a median difference of 2 (IQR 1-2.5) vs in person. Higher scores were not favored by either mode of assessment. Three participants received inconsistent cognitive classifications in both groups.
	Tam et al [34], 2017 Community Retrospective chart audit	Median age of 67 years, patients needing perioperative medicine consultations 229 patients	Videoconference Communication technologies, synchronous, pilot N/A	Characterize last-minute cancellations among patients who needed perioperative medicine videoconference consults	Findings—7 (3.1%) patients experienced last-minute cancellations; medical reasons were attributed to 1.3% of these, consistent with international average.
	Wade et al [35], 2015 RACF Direct observation at project sites, semistructured interviews, and video call data	RACF residents, needing general care 3 GP^f^ practices and 3 RACFs	Videoconference Communication technologies, synchronous, pilot N/A	Enable faster access to medical care and avoid unnecessary hospital transfers	Improved (no comparator)—3 of the 40 video consultations were judged by the GPs to have avoided hospital attendance.
	Towers et al [36], 2014 Community, mixed methods evaluation study	Mean age of 77 years, 82% aged ≥70 years, needing general care, especially medicine management 46 clients	Videoconference Communication technologies, synchronous, mature N/A	Improve access to community-based patient care, especially medicine management	Intervention feedback—BEIP model of care was safe for medicines management. Age is not a barrier to technology—clients’ confidence regarding use of technology improved from 34% before starting the project to 81% at the conclusion. Staff also had positive impressions. Lower costs—savings in travel times for clients, as seen in the reduction in visits or travel time from 4.2 hours per week (face-to-face and travel time) to 2 hours with BEIP. There was an increase in time spent directly on medication management from 72% to 90%, corresponding to a reduction in travel time from 28% to 10%.
	Taylor et al [37], 2015 Community Mixed methods evaluation study	Older people defined as ≥65 years of age, needing community-based palliative care and home-based rehabilitation services N/A	Videoconference Communication technologies, synchronous, pilot N/A	Improve palliative care patients living in the community and home-based rehabilitation services for the older adults at home	Noninferior (no comparator)—the effectiveness of videoconference was judged by clinicians as equivalent to or better than a home visit on 192 of 268 (71.6%) occasions.
	Theodoros et al [38], 2016 Community Randomized controlled trial	Mean age of 71 years, needing Parkinson disease rehabilitation 52 patients	Videoconference Communication technologies, information and data sharing, synchronous, pilot Face-to-face consultation	Enable speech treatment for patients with Parkinson disease at home	Noninferior—comparable clinical and quality-of-life outcomes for both intervention and comparator. Significant improvement posttreatment was achieved for several acoustic, perceptual, and quality-of-life measures across the groups.
	Jones et al [39], 2017 RACF Mixed methods evaluation study	N/A; targeted older adults in RACFs with various conditions 6 RACFs and 20 GPs	Videoconference Communication technologies, synchronous, pilot N/A	Improve access to care	Noninferior (no comparator)—most (83%) clients had their needs completely met during the video consultation and did not require additional follow-up.
	Dorsey et al [40], 2017 RACF Gray literature—observational report	N/A; targeted older adults in RACFs with various conditions 4 local RACFs with 200 beds and 30 GPs in 5 practices	Videoconference Communication technologies, synchronous, pilot N/A	Improve timeliness of care; reduce after-hour calls and hospital transfers	Improved (no comparator)—30% of video consultations have prevented hospital admissions and transfers to emergency departments.
	Ward et al [41,42], 2012 and 2014 Community Questionnaire evaluation	Mean age of 67 years, with or without dysphagia 100 patients	Videoconference and self-measurement device (multi-inputs and outputs) to complement the live remote sessions Communication technologies, information and data sharing, synchronous, pilot Face-to-face consultation	Allow for alternative, valid, and reliable clinical swallow examinations	Noninferior—acceptable levels of agreement were observed between raters for the primary outcomes (decisions regarding oral or nonoral intake and safe food and fluids) and over 90% of the clinical swallow examination items.
	Hwang et al [43] (pilot), 2017, and (cost analysis) [44], 2019 Community Randomized controlled trial	Mean age of 67 years, has chronic heart failure 53 total—24 intervention and 29 control	Videoconference 12 weeks, 2× a week 60-minute sessions, and self-measurement device (multi-inputs and outputs) Communication technologies, information and data sharing, synchronous, mature Standard care (center-based rehabilitation program)	Provide rehabilitation in the home for this population	Noninferior—no significant between-group differences on 6-minute walk distance gains after 12 weeks. The secondary outcomes indicated that the experimental intervention was at least as effective as traditional rehabilitation. No significant differences in quality-adjusted life years between the 2 groups. Lower costs (cost saving)—total health care costs per participant were lower in intervention group (US $1067) after 6 m. The ICER^g^ adopting the health care provider’s perspective was (US $2789) per QALY^h^ gained.
	Katalinic et al [45], 2013 Community Technical issue evaluation and satisfaction survey	Mean age of 66 years undergoing cardiac coaching 102 patients	Videoconference Communication technologies, synchronous, pilot N/A	Improve access to services, self-management of health conditions and health education, and to reduce social isolation	Intervention feedback—both patients and clinicians readily accept and learn how to use new technologies, particularly where using them saves significant amounts of time. Age does not appear to be a barrier. Device installations required substantial resources and would not be suitable for high-turnover situations. The lack of broadband internet in some regional and rural areas was a barrier. Initial concerns that equipment would be lost or stolen from patient’s homes were unfounded. Higher costs—owing to capital cost and recurrent monthly costs of the tablet for patient use. However, if patient or a family member already owned a tablet, Android device, or PC, then the team was able to load the videoconference software onto it at no cost.
**Phone-based**					
	Beauchamp et al [46], 2020 Community Randomized controlled trial	Older women aged 50-74 years, Italian or Arabic speaking and at risk of breast cancer 195 total—95 intervention and 100 control	Phone-based screening reminder Communication technologies, synchronous, pilot No phone call reminder	Improve breast cancer screening rates for older women	Improved incidence ratio (95% CI) of booking was 10.1 (3.9-26.3) times higher among Italian women, and 11.6 (2.9-46.5) among Arabic women in the intervention than no reminder.
	Almeida et al [47], 2021 Community Randomized controlled trial	Older adults aged ≥65 years at risk of depression 307 total—154 intervention and 153 control	Scheduled phone-based support (45 min, 3 sessions) Communication technologies, synchronous, pilot Nonscheduled support	Reduce depressive and anxiety symptoms among older people with subthreshold depression living in regional and remote areas of Australia	Improved odds ratio of depression associated with the intervention was 0.49 (95% CI 0.04-3.49)—blind assessment. Intention‐to‐treat analyses found modest nonsignificant effects of intervention, whereas complete‐case analyses showed improvements in depression and anxiety symptoms over 52 weeks and mental health quality of life compared with control.
	Cameron-Tucker et al [48], 2016 Community Randomized controlled trial	Mean age of 69 years with chronic obstructive pulmonary disease 65 total—35 intervention and 30 control	Phone-based coaching and support (8-12 weeks) Communication technologies, synchronous, pilot Waitlist control	Meet clinical demand for pulmonary rehabilitation. Support participants in undertaking exercise and address other mutually identified health behaviors	Worse—increase in median 6-minute walk distance of 12 (39.1) m in controls only, no change in intervention group.
	Lahham et al [49], 2020 Community Randomized controlled trial	Mean age of 68 years with chronic obstructive pulmonary disease 58 total—31 intervention and 27 control	Phone-based support (1 home visit and 7 calls weekly) Communication technologies, synchronous, pilot Standard care	Improve exercise capacity in people with mild COPD^i^	Noninferior—both groups showed improvements in exercise capacity, symptoms, and health-related quality of life over time. No difference in 6-minute walk distance, but participants were more likely to have clinically important improvements in emotional function at end-intervention or 6 months.
	Lannin et al [50], 2013 Community Randomized controlled trial	Mean age of 68 y with stroke 559 total—282 intervention and 277 control	Phone-delivered health questionnaire (3-6 m postevent) Communication technologies, synchronous, pilot Mail-delivered health questionnaire	Improve efficiency and define differential costs of telephone- vs mail-based assessments of outcome	Improved—shorter time needed to complete follow-up (mean difference: 24.2 days vs comparator). Higher costs—the average cost of completing a telephone follow-up was greater (US $20.87 vs US $13.86 per patient) and had a similar overall response to the mail method (absolute difference: 0.57%). This was attributable to cost of salaries of staff employed to undertake the telephone calls, often on multiple occasions.
	Hullick et al [51] (implementation), 2020, and Ling et al [52] (cost analysis), 2019 RACF (Hullick et al [51])—a stepped wedge nonrandomized cluster trial with 11 steps implemented from May 2013 to August 2016 (Ling et al [52])—14 weeks of ACE^j^ and emergency service data (June-September 2014)	RACF residents, needing acute care 920 call records	Phone-based triage (telephone support, evidence-based algorithms, defining goals of care for emergency transfer, case management in emergency department, and an education program) Communication technologies, synchronous, pilot Standard care—as determined by the RACF. When a resident deteriorates, the primary care physician may or may not be contacted	Improve the capability of aged care facilities to manage acutely unwell residents and reduce avoidable emergency department presentations by aged care residents	Improved—statistically significant reduction in hospital admissions (12% to 10%) and 7-day emergency visit rates (5.7% to 4.9%, 981 saved presentations). Standardized numbers of total calls per 100 beds decrease by implementation level: from 34.4 calls per 100 beds for high implementers, and 24.1 and 17.9 per 100 beds, respectively for medium and low implementers. High implementers had the lowest rate of emergency presentations at 29.5 per 100 beds. Lower costs—compared with standard care, intervention saved an estimated Aus $921,214 (US $625,159). Per 100 RACF beds, savings are Aus $15,513 (US $10,406) for ambulance and Aus $6638 (US $4450) for emergency departments. Level of savings increased positively with implementation level; Aus $26,924 (US $ 18,049) per 100 beds for high implementer, and Aus $14,083 (US $9440) and Aus $8692 (US $5826) for medium and low implementers, respectively.
	Doyle et al [53] (trial), 2017, and Moayeri et al (cost analysis) [54], 2019 Community Randomized controlled trial and cost analysis	Mean age of 68 years with chronic obstructive pulmonary disease and comorbid depression and anxiety 110 total—54 intervention and 56 control	Phone-based cognitive-based therapy (8 scheduled sessions, once a week for 30 minutes) Communication technologies, synchronous, pilot Placebo-befriending (control arm) program	Provide nondirective emotional social support in COPD participants with mild to severe depression or anxiety.	Improved—significant improvement in anxiety symptoms for the befriending group at week 9 and week 17 follow-up, with a small to medium effect size (Cohen d=0.3). Improvement in depression symptoms for both groups was only significant for intervention group at week 17 (Cohen d=0.4). No differences were found in quality of life. Lower costs (and may be cost-effective)—incremental cost of Aus $407 (US$273), plus a negative, nonsignificant incremental QALY gain of −0.008 per patient compared with control. Mean incremental cost-utility ratio was US $33,717 (Aus $50,284) cost saving per QALY sacrificed. Assuming willingness-to-pay threshold of Aus $64,000 (US $42914), the probability of intervention being cost-effective was 42%.
	Price et al [55], 2018 Community Retrospective analysis	Mean age of 69 years, Italian and Greek speaking older adults with cardiac conditions 383 total—82 intervention and 301 English-speaking control (in late 50s)	Phone-based coaching and support to manage risk factors (1-week postevent, calls every 4-6 weeks until deemed capable of self-managing risk factors) Communication technologies, synchronous, pilot English-speaking population	Extend telephone cardiac coaching to CALD^k^ (Greek and Italian) populations	Noninferior—individuals met all target variables other than HDL^l^ cholesterol. At least 5% body weight loss was achieved by 50% of English cohorts, 98% of Italian cohorts, and 53% of Greek cohorts. The Italian cohort was less likely to achieve weight target (OR 0.3 vs English cohort) but more likely to meet the waist target (OR 4.8). The Greek cohort was less likely to meet the waist target (OR 0.2) but no difference regarding the weight target.
	Regan et al [56], 2017 Community Randomized controlled trial	Older adults aged ≥65 years, living in community targeted for influenza vaccine 3613 total—1781 intervention and 1832 control	SMS text messaging reminder for vaccination Communication technologies, asynchronous, pilot No SMS text message reminder	Increase uptake of seasonal influenza vaccine	Improved—in ≥65-year -old group, 20.5% were vaccinated vs control at 15.8% (OR 1.26).
	Sampurno et al [57], 2016 Community test-retest reliability analysis	Older men aged 55-75 years with prostate cancer 168 patients	Phone-delivered health questionnaire Communication technologies, synchronous, pilot Self-administered health questionnaire	Alternative way to evaluate QoL^m^ for men with prostate cancer	Noninferior—kappa-linear model resulted in a moderate agreement across the urinary or bowel or sexual bother scores for both modes of administration; with greatest concordance recorded for bowel bother (90%).
	Tang et al [58], 2020 Community Randomized controlled trial	Mean age of 78 years, older adults with age-related macular disorder 155 total—77 intervention and 78 control	Phone-based nutrition coaching and support (20 minutes per month for 4 months) Communication technologies, synchronous, pilot Off-the-shelf brochures	Improve dietary behaviors among patients with AMD^n^	Noninferior—at 3 months after the intervention, there was no difference in participants meeting the dietary goals nor in intake of total vegetables (primary outcomes). There was a significantly higher intake of nuts, dark green leafy vegetables, and legumes and reduced intake of sweets and processed or prepared foods (secondary outcomes) in intervention vs control.
	Tutty et al [59], 2019 Community A survey of feasibility and acceptability and a cost analysis	Mean age of 67 years, women with breast cancer gene variant 107 patients	Phone-based genetic counseling (0.25 hours each before counseling and after counseling) Communication technologies, synchronous, pilot In-person genetic counseling	Evaluate TGC service for access, acceptability, effectiveness, and equity	Noninferior—while the impact of testing was greater for those with a positive test result, phone genetic testing did not put the additional psychosocial burden on participants. Lower costs—lower median time spent on phone genetic counseling, majority of costs arose from shipping and delivery for blood samples. The median per-patient cost was US $61 (Aus $91.52) compared with US $72 (Aus $107) for control. Total cost to identify variant affecting function was Aus $1000 (US $670) per person for intervention vs Aus $1173 (US $786) for comparator.
	Voukelatos et al [60], 2015 Community Randomized controlled trial	Mean age of 73 years with high risk of falls 385 total—191 intervention and 194 control	Phone-based coaching and support (48 weeks, 2 calls in the first stage and one-half-way) + manual for walking program Communication technologies, synchronous, pilot Control intervention—participants received health information unrelated to falls	Prevent falls in frail older adults	Noninferior—no difference in fall rates between intervention and control in the follow-up period (incidence rate ratio=0.9). At the end of study, intervention group spent more time exercising in general and specifically walking for exercise (median 1.7 vs 0.8 hours per week).
	Walters et al [61], 2013 Community Randomized controlled trial and semistructured interviews	Mean age of 68 years, older adults with chronic obstructive pulmonary disease 182 total—90 intervention and 92 control	Phone-based coaching and support (16 calls tapering to every 2 months, over 12 months) Communication technologies, synchronous, pilot GP care and noninterventional brief phone calls	Improve QoL in patients with COPD, adopt, and maintain healthy behaviors	Noninferior—no difference in quality of life and chronic obstructive pulmonary disease admission between groups, but self-management capacity increased in intervention group (knowledge domain). Anxiety decreased in both groups and coping capacity improved.
	Young et al [62], 2018 Community Prospective pre-post evaluation design	Mean age of 82 years, needing nutritional discharge planning from inpatient care 80 total—41 intervention and 39 control	Phone-based nutrition discharge planning and dietetic follow-up (for 4 weeks after discharge) Communication technologies, synchronous, pilot Standard care (before the intervention)	Improve nutritional and functional recovery in malnourished or high malnutrition risk older patients	Noninferior—no difference in nutritional status, although the intervention cohort maintained weight while preintervention cohort lost weight. Greater improvement in gait speed in intervention group. Across both cohorts, half were readmitted to hospital and 10% died within 12 weeks after discharge, but length of hospital stay was shorter in the intervention group.
	Hammersley et al [63], 2015 Community Prospective cohort study	Mean age of 70 years, older adults with risks of 2 or more chronic disease 250 patients	Phone-based behavior change coaching (5 calls+1 follow-up call 12-18 months after the program) Communication technologies, synchronous, pilot N/A	Improve BMI and physical activity	Improved—significant improvements in BMI and weight and increased average number of minutes spent in moderate to vigorous intensity physical activity per week by 157 minutes. At follow-up, 86% of participants maintained or further improved their health behavior.
**Web-based**					
	Alley et al [64], 2018 Community Randomized controlled trial	Mean age of “older adults” (as per study)—64 years from the general population 504 total—165 in intervention 1, 168 in intervention 2, 171 in control; 205 older people	Web-based informational website and self-management program (web 2.0 and 1.0) and pedometer Information and data sharing, asynchronous, pilot Paper logbook	Improve engagement and effectiveness of WALK 2.0 program in older adults	Improved—modified informational website (intervention 2, “Web 2.0”) had higher levels of website engagement and physical activity changes compared with the web 1.0 (older version) group at 3 months but not at 12 and 18 months (not older adult specific). Web 2.0 was more effective than the logbook control at 3 months, and this effect was significantly stronger in older than younger adults.
	Burns et al [65], 2013 Community pre- and postintervention, repeated measures design	Older adults defined as >55 years, with asthma 51 matched pre- and posttest data	Web-based informational website and self-management program (AsthmaWise) Information and data sharing, asynchronous, pilot Preintervention	Provide asthma self-management program developed for older Australians	Improved—78.4% reported knowing more about how to manage their asthma, 49% experienced an improvement in their asthma symptoms. Asthma knowledge, asthma control, and asthma quality of life were all seen to significantly improve. Scores for the 3 subscales, breathlessness, mood, and social also showed significant improvements.
	Kiropoulos et al [66], 2011 Community Randomized controlled trial	Mean age of 65 years, Greek- and Italian-born immigrants 202 total—110 intervention and 92 control	Web-based informational website and self-management program (MIDonline website) Information and data sharing, asynchronous, mature Control intervention—semistructured interview with a bilingual interviewer	Increase depression literacy or reduce depression stigma and depressive symptoms in CALD population	Improved—those in intervention displayed higher depression literacy scores post assessment and at follow-up than control and showed a significantly greater decrease in mean personal stigma scores post assessment and at follow-up. For perceived stigma, there was no significant difference. For level of depression, there was no significant difference.
	O’Moore et al [67], 2018 Community Randomized controlled trial	Older adults defined as >50 years, with knee osteoarthritis 69 total; 44 intervention and 25 control	Web-based self-education program (iCBT^o^ Sadness Program) Information and data sharing, asynchronous, mature Standard care for osteoarthritis	Reduce depressive symptoms and psychological distress and improve overall mental health, self-efficacy, osteoarthritis-related pain, and physical function	Improved—intention-to-treat analyses indicated between-group superiority of intervention over control on the primary outcomes (self-reported depression severity and general psychological distress), at postintervention and 3-month follow-up, and on secondary osteoarthritis-specific measures (pain, stiffness, and physical function) at the 3-month follow-up. Most intervention participants (84%) no longer met diagnostic criteria at 3-month follow-up.
	Staples et al [68], 2016, and Titov et al [69], 2016 Community Randomized controlled trial	Older adults defined as aged >60 years, with anxiety and depression 949 for effectiveness trial; 433 for clinician-guided and self-guided comparative trial	Web-based self-education program (Wellbeing Plus Course) in the clinic or real-world setting Information and data sharing, asynchronous (but intervention may involve direct contact with health care professional), mature Same intervention but provided in research setting (Staples et al) and initial clinician interview followed by self-guided treatment (Titov et al [69])	Intervention for older adults with anxiety or depression	Noninferior—all groups showed significant symptom reductions at posttreatment. Results were maintained at 3-month follow-up. Within-group symptom changes were comparable. Initial symptom severity was higher in the clinic group and course completion was lower.
	Titov et al [70], 2015 Community Randomized controlled trial	Mean age of 65 (range 61-76) years, older people with symptoms of depression 54 total; 29 intervention and 25 control	Web-based self-education program (iCBT) Information and data sharing, asynchronous, pilot Delayed-treatment waitlist control	Improve access to treatment for older adults with depression	Improved—significantly lower scores on the depression symptom and anxiety in treatment group than control at posttreatment. The treatment group maintained lower scores at 3-month and 12-month follow-up. The treatment group had slightly higher quality-adjusted life years than the control group after treatment. Higher costs (but cost-effective)—costs up by US $35 and an incremental cost-effectiveness ratio of US $2947.
	Torrens et al [71], 2017 Community cross-sectional study	Older adults included, comprise 2 groups—60-74 and ≥75 years in the general population 2,074,800 electronic health record registrations from July 1, 2012, to February 18, 2015	Electronic health record (My Health Record) Information and data sharing, asynchronous, mature N/A	Understand differential uptake of My Health Record; older adult data extracted	Intervention use—registrations decreased as the population became older; 60-74 years: male 8.7%, female 9.6%; ≥75 years: male 9.4%, female 6.7%. Some population groups that already experience health inequalities were underrepresented in the registration pool at the time of this study, such as men and older women.
	Vandelanotte et al [72], 2012 Community Prospective web-based study	Mean age of 67 years, from the general population 803 total; 235 aged 60-89 years	Web-based self-management program Information and data sharing, asynchronous, pilot N/A	Alternative physical activity intervention	Improved—old age group engaged in significantly more total physical activity mins than young age group and middle age group. On average, all age groups increased their weekly total physical activity minutes and the number of total physical activity sessions significantly over time from baseline to 1-month follow-up (+31 minutes/+1.2 sessions).
	Wilson et al [73], 2015 Community Randomized controlled trial	Older adults aged 50-74 years with bowel cancer 3408 total; 1137 in intervention 1 ITT^p^, 1136 in intervention 2 and 1135 control	Web-based self-education program, tailored and nontailored Information and data sharing, asynchronous, mature Mail-delivered control	Increase bowel cancer screening participation	Noninferior—no significant difference in ITT group for return of bowel cancer screening kit. Age was positively associated with kit return. Participants not wanting to screen at baseline were significantly more likely to decide to screen and return kit than control. Analysis of salience and coherence of screening and self-efficacy were improved, and fecal aversion decreased by tailored messaging.
	Staffieri et al [74], 2011 In-hospital or clinic Prospective study	Older adults aged >65 years with primary open-angle glaucoma 133 patients	Electronic health record Information and data sharing, asynchronous, pilot N/A	Increase screening of high-risk individuals for undiagnosed glaucoma	Improved—a telemedicine model is an efficient method for screening, grading, and notifying participants of examination results. For every 19 participants screened, 1 new case of previously undiagnosed case of glaucoma was identified. Targeted screening for glaucoma increases the yield of identifying individuals with undiagnosed glaucoma or those at greatest risk.
	Cadilhac et al [75], 2020 Community Randomized controlled trial	Mean age of 68 years, stroke patients 54 total—25 intervention and 29 control	Web-based database, tailored SMS messaging or email messaging system, and standard care Communication technologies, remote monitoring, information and data sharing, asynchronous, pilot Standard care	Assist person-centered goal setting (educational and self-management) for stroke	Noninferior—at follow-up, goal attainment in the intervention group was achieved for goals related to function, participation, and environment (control: environment only), and nonsignificant improvements for most self-management domains (eg, social integration and support) and several quality-of-life domains mainly in intervention group. No unintended harms or effects were reported. Low-cost intervention—824 electronic messages (446 SMS text messages; 378 emails) were sent during the intervention period (657 intervention; 167 control), with a total cost of US $26 or 4.7 cents per message sent.
**Application**					
	Bhattarai et al [76], 2020 Community semistructured interview	Mean age of 73 years, 89% female with arthritic pain 16 patients	App Information and data sharing, asynchronous, pilot N/A	Self-management activities to adequately manage pain	Intervention feedback—participants enjoyed the accessibility of the app. The app provided pain self-management instructions, helped diarize self-management plan, and assisted with monitoring progress and planning. Challenges were vision-related when engaging with the app on a small screen, and there were issues of poor dexterity and agility of arthritic fingers. Some expressed concerns that this could lead to overfocus on pain and catastrophizing behaviors.
	Thomas et al [77], 2020 Community cross-sectional (web-based survey)	General participants in community, excluding health care professional or if one had been tested for COVID-19, older people included 227 aged 65-74 y (15%); 154 aged ≥75 y (10%)	App Information and data sharing, asynchronous, pilot N/A	COVID-19-related tracking system	Intervention use—65-74 years, 97 (42.7%) downloaded, 28 (12.3%) intend to download, 65 (28.6%) refused to download, and 37 (16.3%) were unsure. For >75 years, 62 (40.3%) downloaded, 28 (18.2%) intend to download, 42 (27.3%) refused to download, and 22 (14.3%) were unsure. Download rate lower than those aged <65 years.
	Tongpeth et al [78], 2020 Community Randomized controlled trial	Mean age was 65 years, patients with acute coronary syndrome 70 total—35 intervention and 35 control	App and standard care Information and data sharing, asynchronous, pilot Standard care (bedside education)	Improve heart attack symptom recognition and reaction	Improved—intervention group had a significant improvement in symptom knowledge, attitudes, and beliefs over the 6‐m period; and no significant improvement in the standard care group participants (58.35% at baseline to 82.72% at 1 month and 83.55% at 6 months). There was higher ambulance use in the intervention group than the standard care group (33.33% vs 18.18%). There was no harm or unintended effects in either group of the study.
	Wonggom et al [79], 2020 Community Randomized controlled trial	Mean age of 68 years, 81% male, 47% have been living with heart failure for >5 years 36 total—19 intervention and 17 control	App and standard care Information and data sharing, asynchronous, pilot Standard care (bedside education)	Improve knowledge and self-care behaviors of patients with HF^q^	Noninferior—at 90 days, the intervention group participants had a higher increase in knowledge score than control group (22.2% vs 3.7%). There was no difference on self-care behavior or health care use.
**Multimode (telemonitoring)**					
	Feros Care [80,81], 2014, and Nancarrow et al [82], 2016 Community Longitudinal study	Mean age of 75 years with chronic disease 181 patients	Multimode—telemonitoring system (multi-inputs and outputs), data inputted into relevant peripheral device, and videoconference as required Communication technologies, remote monitoring, information and data sharing, combination of synchronous and asynchronous systems, pilot N/A	Provide complementary virtual health service model for seniors (remote monitoring and improve access to care)	Improved—48% participants reported that they better managed their own health and had better information about their health and improvement in their levels of self-efficacy for managing their chronic disease and other health behaviors. Over the duration of the pilot, participants reported fewer visits to the doctor, emergency department at the local hospital, non–local hospital admissions compared with the preceding year, but no statistically significant reduction in local hospital admissions.
	Tieman et al [83], 2016 Community Prospective cohort qualitative and quantitative study	Mean age of 72 years, needing palliative care 43 patients	Multimode—self-reporting via electronic diary, website with resources, and structured and alert-initiated videoconference Communication technologies, remote monitoring, information and data sharing, combination of synchronous and asynchronous systems, mature N/A	Improve community-based palliative care for patients, carers, and clinicians	Feasible—patients and carers were able to use the technology and did self-report using the apps. There were 611 alerts arising from changes in performance score across the study and 4386 alerts generated through symptom assessment scale. Self-reported data entered by patients and carers did identify changes in performance state and in symptom distress, triggering alerts to the service provider. Scheduled videocall contacts and contacts made in response to triggers led to changes in care.
	De San Miguel et al [84], 2013 Community Randomized controlled trial and interview	Mean age of 73 years, with chronic obstructive pulmonary disease 71 total—36 intervention and 35 control	Multimode—self-reporting via telemonitoring system (multi-inputs and outputs), data inputted into relevant peripheral device, data transmitted to web portal for monitoring Communication technologies, remote monitoring, information and data sharing, combination of synchronous and asynchronous systems, pilot Standard care	Remote monitoring and self-management for COPD	Noninferior—intervention group had fewer emergency presentations and hospital admissions and a reduced length of stay vs control but not statistically significant. No change in quality of life but a clinically significant change found for the mastery domain between baseline and 6 months. Cost saving—the reduction in health service use was large enough to result in significant cost savings (equipment costs and labor costs), with the annual cost savings of intervention group of US $1968 per person vs control.
	Ding et al [85], 2020 Community Randomized controlled trial	Mean age of 70 years, with chronic heart failure 184 total—91 intervention and 93 control	Multimode—device (tablet) and app-based automated monitoring system (weight input), phone-based support and modified standard care Communication technologies, remote monitoring, information and data sharing, combination of synchronous and asynchronous systems, pilot Modified standard care	Remotely support self-management of chronic heart failure and evaluate compliance	Noninferior—nonsignificant increase in compliance criterion of weighing at least 4 days per week in intervention vs control group but significantly higher stricter compliance standard of at least 6 days a week was met and a significantly improved score in health maintenance, medication adherence, and diet. Quality of life, hospitalizations, or emergency presentations were not significantly different.
	Elliot Bereznicki et al [86], 2013 Community Prospective study and survey	Mean age of 70 years, needing warfarin therapy 168 in survey (69.1% participation rate); 22 in trial	Multimode—medication self-testing, data entered into the website (educational resources also available) plus physician-led custom system monitoring using CoaguChek XS monitor Communication technologies, remote monitoring, information and data sharing, combination of synchronous and asynchronous systems, pilot Standard care (laboratory testing and physician dosing)	Improve warfarin therapy (specifically, international normalized ratio) self-monitoring	Noninferior—CoaguChek XS prothrombin time international normalized ratio values were significantly correlated with laboratory values. There was a statistically significant improvement in the time in therapeutic range in intervention group. No clinical outcomes (events of major bleeding or thromboembolism) were observed.
	Karunanithi et al [87], 2018 Community Collective report of pilot studies for Smarter Safer Homes program	Older adults aged >65 years, living alone 17 in pilot number 1 and 10 in pilot number 2	Multimode—app-controlled home sensors, device (tablet) and web portals (Smarter Safer Home platform) Communication technology, remote monitoring, information and data sharing, asynchronous pilot N/A	Consumer-directed age care reform for remote monitoring	Feasible—the platform demonstrated the value of SSH platform to switch from the default passive to close and real-time monitoring of residents for those vulnerable and the sleep monitoring data to determine the resident’s well-being.
	Wade et al. [88], 2012 Community quasi–randomized controlled trial	Mean age of 81 years, with chronic disease and at risk of being admitted to residential aged care facilities 43 patients	Multimode—telemonitoring system (multi-inputs and outputs) and device (pendant alarm), readings outside the set parameters were faxed to client’s GP Remote monitoring, information and data sharing, asynchronous, pilot Standard transitional care	Understand acceptance and usage of videoconference and telecare products by frail older clients	Feasible—there was a 13% videoconference reading failure rate. There was no significant difference between clients with and clients without carers for the reading failure rate. There was no significant difference between clients with a carer and without a carer.
	Schoene et al [89-92], 2011, 2013-2015 Community Randomized controlled trial and cross-sectional study	Older adults >70 years of age, frail with high risk of falls 18 in pilot; 90 total in trial—47 intervention and 43 control	Multimode—dance mat-based app plus automated data transmission to clinicians Communication technology, remote monitoring, information and data sharing, asynchronous pilot Standard care	Measure fall risk and deliver exercise-based intervention into the homes of older adults	Improved—enhanced stepping reaction times, reduced physiological measure of fall risk, and improved timed up and go test involving cognitive demand. In the larger trial, authors extended the range of exercise-based games that could be delivered through the system, resulting in further improvement in measures of processing speed, visuospatial ability, and concern about falling. Test-retest reliability of the dance mat response time was high.
	Department of Veterans’ Affairs [93], 2017 RACF/community Implementation study and cost-effectiveness analysis	Mean age of >70 years, with one or more of 4 chronic conditions (coronary artery disease, chronic obstructive pulmonary disease, chronic heart failure, or diabetes) 250 patients	Multimode—telemonitoring system (multi-inputs and outputs), data inputted into relevant peripheral device and transmitted to portal at practice, videoconference as needed Communication technologies, remote monitoring, information and data sharing, combination of synchronous and asynchronous systems, pilot Standard care	Remote monitoring and improve management of chronic conditions	Improved—a small reduction in public hospital admissions and clinical complexity of both public and private hospital episodes for some participants, smaller increases in the use of general practice health services vs control, and improved quality of care through earlier identification of health issues and medication management. Participants improved health literacy and self-management, leading to a more cooperative approach to health management, improved relationships between participants and their practice, and an overall improved sense of assurance and well-being and helped delay entry into residential aged care facility. Cost-effective (but not for entire cohort)—an overall cost-effective analysis based on the operation of the trial model, excluding trial set-up costs, showed operation as a service in the future could be cost neutral. Other qualitative analysis indicated that value for money is more likely to be achieved as a short to medium-term intervention for appropriate participants in appropriate clinical settings.
	Celler et al [94], 2016 Community before-after, case-matched prospective study, cost-effectiveness analysis	Mean age >68 years, home-based 288 total—intervention 114 and 173 control	Multimode—telemonitoring system (multi-inputs and outputs), data inputted into relevant peripheral device, monitored by champions and liaised to health care providers Communication technologies, remote monitoring, information and data sharing, combination of synchronous and asynchronous systems, pilot Standard care	Improve management of care and remote monitoring	Improved—53.2% reduction in the rate of admission to hospital (reduction of 0.22-1.0 hospital admissions), 75.7% reduction in the rate of length of stay (reduction of 7.3-9.3 days) and >40% reduction in mortality. Lower costs—46.3% reductions in rate of Medicare Benefits Schedule expenditure (savings US $410-US $441) and 25.5% reduction in rate of Pharmaceutical Benefits Scheme expenditure (savings US $30-US $238). Analysis of this model suggests that for chronically ill patients, an annual expenditure of US $1853 could generate a saving of between US $11,000 and US $12,934 per annum, representing a return on investment of between 4.9% and 6.0%.
	Halcomb et al [95], 2016 Community Pre- and posttest design	Mean age of 81 years, with chronic disease 29 patients	Multimode—telemonitoring system (multi-inputs and outputs), device prompts patients to undertake monitoring, data inputted into relevant peripheral device and results securely provided to health care provider Communication technologies, remote monitoring, information and data sharing, combination of synchronous and asynchronous systems, pilot N/A	Health monitoring of older people with chronic disease	Improved—57.1% (n=12) participants agreed that telemonitoring provided them with a sense of security and peace of mind, assisted them to manage their health (n=11, 52.4%), and had improved confidence in managing their care. Nearly two-thirds of participants felt more involved in their health care (n=14, 66.7%) and had better understanding regarding changes in their condition.
	Chow et al [96], 2018 Community Retrospective analysis of biometric and self-assessment readings	Older adults (aged 44-87 years; majority >65 years) living in isolated rural area 24,545 data points from 2932 readings	Multimode—telemonitoring system (multi-inputs and outputs), data inputted into relevant peripheral device and transmitted to health care provider Communication technologies, remote monitoring, information and data sharing, combination of synchronous and asynchronous systems, pilot N/A	Assess quality of data collected and describe events, and obtain further information to support future research and implementation of telemonitoring in South Western Sydney	Events identified—over half showed high clinical risk; 93 occasions required GP escalation, 23% (n=14) for respiratory conditions. Nine were hospitalized, 51% of these for respiratory conditions.
**Mixed evaluation**					
	Pasalich et al [97], 2013 Community Prospective study and survey	Insufficiently active 60- to 70-year olds, mean age of 65 years 374 total—176 intervention and 198 control	Mixed evaluation—booklet, calendar, exercise chart, newsletter, device (pedometer), and phone- and mail-based support Communication technology, remote monitoring, information and data sharing, combination of synchronous and asynchronous systems, pilot Same intervention model but in regional hospital (already established before study)	Improve diet and physical activity levels for seniors	Improved—intervention significantly increased fat avoidance behaviors, and there was a nonsignificant increase in strength exercises, fiber intake, BMI, and waist-to-hip ratio either after the program or at follow-up compared with control.
	Wootton et al [98], 2019 Community Randomized controlled trial, multicentered	Mean age of 70 years, older people with chronic obstructive pulmonary disease seeking to improve physical activity 86-33 intervention and 29 control	Mixed evaluation—phone-based support and biofeedback via pedometer (worn 7 days on 3 occasions over 14 months) Communication technologies, remote monitoring, information and data sharing, combination of synchronous and asynchronous systems, pilot Standard care	Improve physical activity	Not effective—no differences in any of physical activity variables from baseline to completion of the program. Ongoing feedback was no more effective than no feedback in improving physical activity.
	Haynes et al [99], 2020 Community semistructured interviews	Participants aged >60 years, mean age of 72, community-dwelling and those who may benefit from physical activity and fall-prevention program 32 patients	Mixed evaluation—home visit, pedometer (self-monitoring) plus phone-based coaching and support (fortnightly for 12 months) Communication technologies, information and data sharing, combination of synchronous and asynchronous systems, pilot Standard care (in-hospital consultation)	Promote physical activity and preventing falls	Improved—most participants reported that the intervention increased physical activity levels, embedded activities, and generated positivity about physical activity. They were motivated by quantified physical activity feedback, self-directed goals, and person-centered coaching. Social connectivity motivated some, but the intervention did not support this well.
	Brickwood et al [100], 2021 Community Randomized controlled trial	Older adults aged >60 years, mean age of 72 years, needing to maintain physical activity or those at risk of having chronic disease 117—37 intervention, 1 (pedometer), 38 intervention, 2 (telephone), and 42 control	Mixed evaluation—pedometer (worn over 12 months) synchronized to smartphone app for biofeedback, or telephone counseling (fortnightly for the first 3 months and monthly for the final 9 months) Communication technologies, remote monitoring, information and data sharing, combination of synchronous and asynchronous systems, pilot Standard care	Assist ongoing support to maintain physical activity levels and health outcomes	Not effective—no difference in overall step counts and quality of life between groups, but telephone and pedometer groups maintained daily step counts, and standard care showed a reduction over 12 months. Unexpected findings included significantly higher diastolic blood pressure in pedometer group than standard care, and 10-time sit-to-stand was significantly slower on the telephone group compared with standard care.
	Jancey et al [101], 2011 Community Randomized controlled trial	Older adults aged 65-74 years, those needing to increase physical activity 231 total—100 intervention and 131 control	Mixed evaluation—a booklet, pedometer (self-monitor), telephone support (2×), call center access for feedback and advice; follow-up at baseline and 6 months (original group) plus 18 and 21 months (booster group) Communication technologies, information and data sharing, synchronous, pilot Standard in-person follow-up care	Increase older adults’ physical activity levels	Improved—time spent walking for recreation (original group only) and errands per week (original and booster groups) were increased. Walking levels for the control group remained stable over the study period.
	Menant et al [102], 2018 Community Prospective study	Older adults >50 years (mean age of 68 years) who reported dizziness in the past year 305 total—154 intervention and 151 control	Mixed evaluation—single or combination of (1) web-based cognitive behavioral therapy, (2) web-based or booklet-based cognitive behavioral therapy support plus telephone support (8 weeks), (3) home-based exercise for 6 months plus home visits and a phone call (at 12 weeks), or (4) medical management Communication technologies, information and data sharing, combination of synchronous and asynchronous, pilot Standard care	Reduce dizziness handicap and self-reported dizziness and enhance balance and gait as needed by the individual	Improved—analyzed as a multifaceted tailored intervention, dizziness scores in the intervention group were reduced versus control. No difference in dizziness episodes, reaction time, and step-time variability during gait. No serious intervention-related adverse events occurred. When analyzed individually, exercise group had a reduced physiological fall risk, and cognitive-based therapy recipients had improved anxiety.
	Williams et al [103], 2012 Community Randomized controlled trial	Mean age of 67 years, older people with concomitant diabetes and chronic kidney disease 80—39 intervention and 41 standard care	Mixed evaluation—home visit, self-monitoring of blood pressure, individualized medication review, 20-minute offline video education and telephone follow-up support (12 weeks), evaluated at 3, 6, and 9 months after the intervention. Communication technologies, synchronous, pilot Standard care (clinical blood pressure management and others as needed)	Improve blood pressure control and medication adherence in adults with coexisting diabetes and chronic kidney disease	Not effective—no difference in medication adherence but mean systolic blood pressure was reduced in the intervention group at 9 months postintervention. Participants enjoyed being more actively engaged in self-management with minimal inconvenience or cost to their routine.
**Secondary virtual care**					
	Padayachee et al [104], 2019 Community Implementation study of allied health–led model of care	Mean age of 79 years, older adults who are hospitalized in Caboolture and Kilcoy (rural Queensland) and need advanced care at a regional hospital 141—93 intervention and 48 control	Secondary—videoconference for a weekly case conference between health care team at the rural hospital or patients and geriatrician at a regional hospital plus on-site geriatric care in the rural center Communication technologies, synchronous, pilot Same intervention model but in regional hospital (already established before study)	Improve patient care in rural centers by improving care flow and management, improve bed occupancy rate, and reduce pressure on regional hospital	Noninferior—the model successfully treated patients effectively and safely. Participants had similar lengths of stay to those cared for in regional hospital, and most were able to be safely discharged home. Cost-effective—cost comparison showed similar outcomes, with similar per bed day costs achieved in regional hospital vs the rural center. Specialist medical input was provided cheaply using videoconference. Use of beds at the rural center to increase from 50% to 80% after 2 years.
	Gallagher et al [105], 2020 Community Randomized controlled trial	Mean age of 65 years, older adults using anticoagulants 72 total—36 intervention and 36 control	Secondary—1 face-to-face education and risk management session (standard care) 4 follow-up telephone calls over a 3-month period Communication technologies, synchronous, pilot Standard care	Ensure appropriate use of oral anticoagulation and improve quality of life, guideline adherence, and cardiovascular risk factor profiles in individuals with atrial fibrillation	Not effective—no significant differences between groups were observed for quality of life. Appropriate use of oral anticoagulation did not differ between groups.
	Clemson et al [106] (trial), 2016; Provencher et al (post hoc) [107], 2020; and Wales et al (economic evaluation) [108], 2018 Community Randomized controlled trial	Mean age of 81 years, frail older adults 400 total—198 intervention and 202 control	Secondary—home visits plus telephone follow-up over 3 months Communication technologies, synchronous, pilot Standard care (in-hospital consultation)	Reduce hospitalizations and difficulty in performing activities of daily living among older adults	Not effective—no difference in prehospital functional status and number of people with unplanned readmissions. Post hoc analyses suggest intervention may reduce unplanned rehospitalization and emergency department presentations at 3 months, but more evidence is needed. Higher cost (and not cost-effective)—ICER of US $41,548 per person with clinically meaningful improvement in activities of daily living. Health services likely would not save money by implementing the program.
	Sharma et al [109], 2017 Community Randomized controlled trial	Mean age of 82 years and malnourished 148 total—78 intervention and 70 control	Secondary—screening and individualized nutrition care referral (standard care) plus telephone follow-up (dietetic counseling monthly for 2 months, 30 min each) Communication technologies, synchronous, pilot Standard care	Early screening to improve clinical outcomes such as length of hospital stay, complication rate, mortality, quality of life and readmission rates	Not effective—no difference in the change in nutrition status, complication rate during hospitalization, quality of life, and mortality at 3 months or readmission rate at 1, 3, or 6 months following hospital discharge. Median total length of hospital stay was 6 days shorter in the intervention group.
	Young et al [110], 2013 Community Randomized controlled trial	Mean age of 68 years, colorectal cancer surgery patients 756 total—387 intervention and 369 control	Secondary—structured phone calls on days 3 and 10 and at 1, 3, and 6 months post hospital discharge based on needs, plus standard follow-up care Communication technologies, synchronous, pilot Standard in-person follow-up care	Improve care coordination and patient-reported outcomes after surgery for colorectal cancer	Not effective—no difference in unmet supportive care needs at all time points, and emergency department presentations or unplanned hospital readmissions at 1 month. Slightly lower unplanned readmission in intervention group. No differences in experience of care coordination, distress, or quality of life at all time points.
	Harrison et al [111], 2011 Community Randomized controlled trial	Mean age of 65 years, underwent surgery for colorectal cancer 75 total—39 intervention and 36 control	Secondary—phone-based support (5 calls 6 months after discharge) and standard follow-up Communication technologies, synchronous, pilot Standard follow-up	Improve quality of cancer care through supportive care	Not effective—clinically relevant but nonsignificant reduction in presentations to emergency departments and readmission to the hospital in intervention vs control (21% vs 33%) and nonsignificant improvement in quality-of-life scores, change scores, and trends.

^a^Older adult characteristic and sample size.

^b^Virtual care intervention, mechanism, maturity, and comparator.

^c^N/A: not applicable.

^d^RACF: residential aged care facility.

^e^CKD: chronic kidney disease.

^f^GP: general practitioner.

^g^ICER: incremental cost-effectiveness ratio.

^h^QALY: quality-adjusted life year.

^i^COPD: chronic obstructive pulmonary disease.

^j^ACE: Aged Care Emergency.

^k^CALD: culturally and linguistically diverse.

^l^HDL: high-density lipoprotein.

^m^QoL: quality of life.

^n^AMD: age-related macular degeneration.

^o^iCBT: internet-based cognitive behavioral therapy.

^p^ITT: intention-to-treat.

^q^HF: heart failure.

### Intervention Delivery

**Figure 1:**
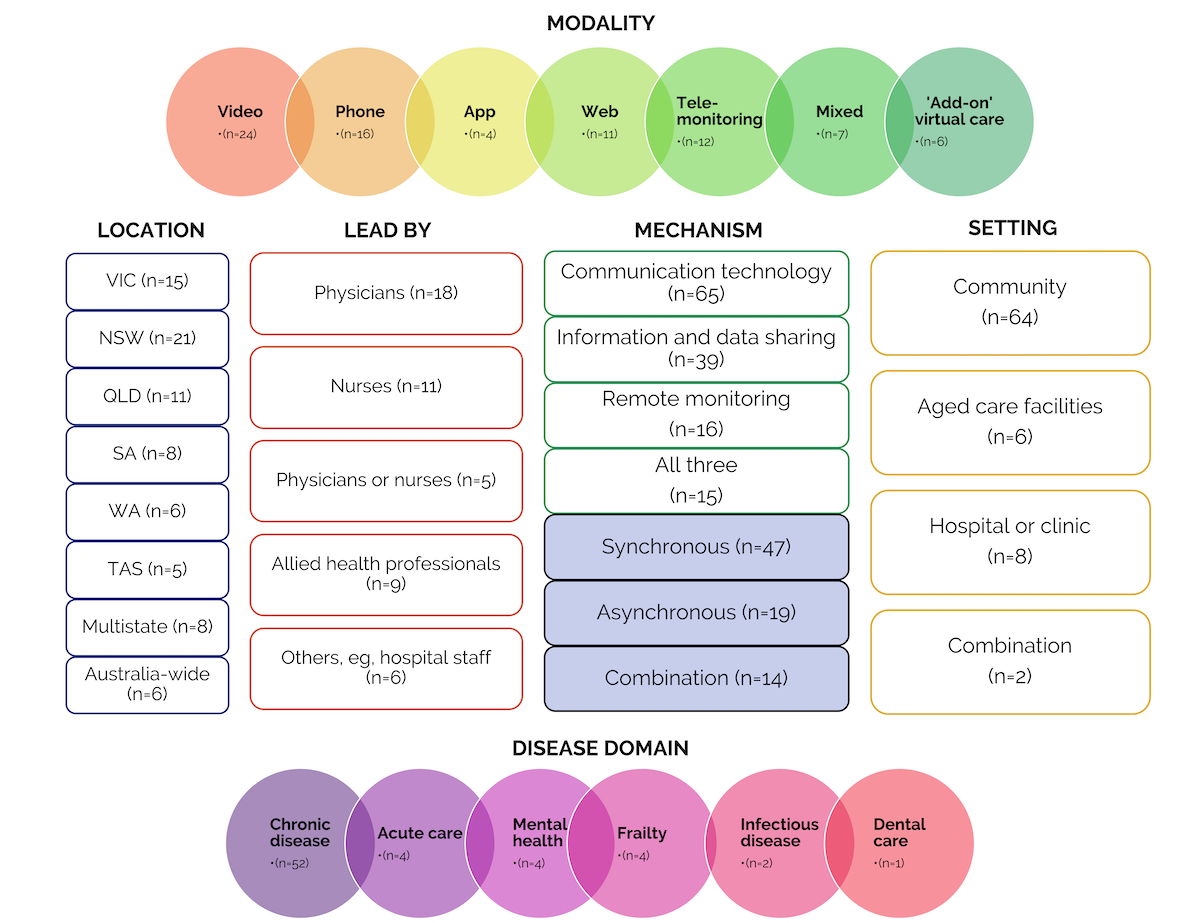
Characteristics of virtual care initiatives for older Australians (n=80), including by type of modality, location, which health care professional leads or has direct involvement with the virtual care, the essential mechanism or function that underpins the initiative (and including whether the mode of delivery was synchronous, asynchronous, or both—shaded in blue), the setting in which the initiatives were delivered, and the disease domain. NSW: New South Wales; QLD: Queensland; SA: South Australia; TAS: Tasmania; VIC: Victoria; WA: Western Australia.

Most initiatives were delivered for community-dwelling older adults at home (64/80, 80%; Figure 1). Six initiatives were for older adults in residential aged care facilities, 8 were delivered in hospital, and 2 included older people in the community, residential aged care facilities, or in-hospital settings. Videoconference (n=28), telephone (n=29), and telemonitoring systems (n=15) were the most commonly used modes of delivery (Figure 1).

Most (n=56) initiatives that involved “communication technology” used synchronous interactions between older people and providers either via the phone (n=28) [46,50,52,54,55,57-62,97,98,101-103,105,107,109-114] or videoconference (n=28) [15-20,22-25,27-30,32-43,45,82], whereas others involved purely asynchronous [56,75] or combined synchronous or asynchronous interactions via the web, app, or other technologies (n=14; Table 1). Only 1 study reported on interactions between health care providers, which was asynchronous [74]. Seven videoconference initiatives required patients to attend a local health care facility to use videoconference equipment [18,19,21-27,29,32].

For initiatives that facilitated “information and data sharing,” 2 involved sharing of medical information from electronic records [71,74], whereas others involved older adults either taking measurements (eg, blood pressure, weight, height, or other physiological data) using devices attached to a portal, which were automatically transmitted to care providers (n=11) [80-82,84,85,87-96] using devices or wearables that automatically recorded and transmitted data (eg, activity trackers; n=2) [98,100], or manually entering data without using any device or wearable (n=2; Table 1) [83,86].

### Intervention Content

A summary of content is provided in Figure 1. Most studies included older adults with or at high risk of having chronic disease (such as heart failure, kidney disease, Parkinson disease, and others, single or multimorbidity; 52/80, 65%; Table 1). The other disease domains included acute care (4/80, 5%), mental health (5%), frailty (5%), and dental care (1/80, 1.3%).

In total, 13 studies used videoconference services specifically to improve access to care [16,19,23,27,28, 32,33,35-37,39,40,104]. Other uses included treatment plan management (n=9) [18,21-26,30-32,38,41,42,111], rehabilitation services (n=3) [20,37,43,44], and social support (from health care personnel; n=2) [15,17]. Telephone initiatives were used predominantly for education, follow-up evaluation, and care support (n=20) [47,50,54,58-62,97-103,105,107,109-113]. Telemonitoring interventions (with or without an additional attached device to measure physiological data) were used to record and monitor progress (n=18) [80,83-89,93-101,103], as alert or reminder systems (n=10) [80,83-88,93,94,96], and for strength training [89-92]. Web-based initiatives were used for treatment or symptom reduction (n=4) [66-70], education and self-management (n=5) [65,66,71,73,75], and support and monitoring (n=3) [64,72,97]. App-based initiatives were used for self-management (n=3) [76,79,103], remote screening [78], and infectious disease tracking [77].

### Effectiveness

Of all identified initiatives, 34 (43.8%) randomized controlled trials and 3 (3.8%) implementation studies evaluated effectiveness for health or behavioral changes compared with in-person care or other suitable comparators.

In total, 28 studies involved the stand-alone delivery of virtual care models with very limited to no face-to-face contact. All but one of these virtual care delivery models yielded either comparable or similar or better health or behavioral outcomes compared with in-person care or other comparators (videoconference: 4/4; telephone: 10/11; telemonitoring: 4/4; web-based: 7/7; app: 2/2). Examples of outcomes measured include emergency visits [51,84,85], hospitalization [51,61,84,85,93], quality of life [25,43,49,53,61,75,84,85], mortality [21,22], physical activity or strength [43,49,60,64,89], health literacy [46,56,58,73,75,78,79,84,85,93], and measures of anxiety or depression [47,53,61,66-70]. Telephone-based coaching was not effective in preventing falls but improved physical activity compared with those receiving unrelated health information [60], while a telephone-based telerehabilitation study noted worse physical activity versus waitlist control [48]. An app-based study reported higher ambulance use; however, this was owing to the improved recognition of heart attack symptoms [78].

In total, 9 virtual care initiatives were delivered as an add-on to standard (in-person) care, only 2 of which reported similar or better outcomes compared with standard care alone. Two initiatives using telephone-based support plus pedometer-based biofeedback, in addition to standard care, resulted in similar physical activity [98,100] and quality of life [100] compared with standard care alone. Only 1 study reported higher physical activity using such an intervention versus standard care alone [101]. Similarly, telephone-based interventions as an adjunct to in-hospital standard care (n=4), home visit (n=1) or various mixed-mode interventions (n=1) did not result in any additional or improved health outcomes [103,107,109], quality of life [105,109-111], and emergency department presentation [107,110,111] and hospitalization compared with standard care alone [107,111]. Incorporating videoconferences in rural centers as an add-on to in-person care yielded similar health outcomes to their regional hospital counterparts [104].

### Cost Assessment

In total, 18 studies reported cost-related assessments (Table 1). Virtual care was associated with lower travel costs for patients [19,26,36] and higher savings for providers from reduced health service use [51,84,93,94]. Two modeling studies of a virtual (telephone-based) emergency department and a remote monitoring initiative indicated that higher implementation rates would lead to more cost-saving effects [51,93]. However, virtual care was associated with high set-up [23,45], maintenance [23,45], and staffing costs [31,50]. Four virtual care initiatives resulted in lower per-patient delivery costs [26,59,75,104] and staff wages [26], while 4 studies reported higher per-patient delivery costs [15,31,50,94]. A virtual dental care initiative demonstrated that remote synchronous (real-time) oral examination was more expensive than face-to-face examination for every aged care facility resident, while an asynchronous review and treatment plan was cheaper than both synchronous and face-to-face delivery modes [31].

Four studies reported the incremental cost-effectiveness ratio (ICER) from the provider’s perspective (Table 1). A videoconference initiative for telerehabilitation was cost saving with an ICER of Aus $4157 (US $2782.57) per quality-adjusted life years gained compared with center-based (in-person) care [44]. Virtual cognitive behavioral therapies yielded an ICER of Aus $50,284 (US $33,665.69) per quality-adjusted life years delivered using telephone compared with in-person befriending [53] and Aus $4392 (US $2940.4) when delivered via the web versus a waitlist control group [70]. Compared with a standard in-hospital consultation, a home visit plus telephone follow-up intervention yielded an ICER of Aus $61,906 (US $41,446) for every older person with a clinically meaningful improvement in daily activities [108].

### User Feedback

#### Technology-Related Issues

Interface-related issues highlighted by older people include a lack of audio or visual clarity [16,24-26,76] and discomfort because of poor dexterity and agility when engaging with virtual care devices [76]. One qualitative study highlighted a lack of consensus regarding the ideal interface, functionality, and size of wearables (pedometers) [99]. In one multimode study, only 54% of patients understood how to access web links provided within database-fed messages [75].

#### Acceptability

Older patients enrolled in the studies found virtual care acceptable (n=22) [16,17,19,20,24-27, 30,31,33,35,36, 58,59,61,65,70,72, 79,94,99,110,112,115], time efficient (n=6) [20,28,30-33,39], and helpful to improve communication with their clinicians (n=8) [15,17,30,31,39,59,61,110,112]. Telemonitoring was often associated with improved self-management (n=4) [84,86,96,115]. A web-based intervention indicated that satisfaction was lower in older people than in younger people [64]. Six studies noted that negative preconceptions (owing to a lack of confidence with technology) were modified with positive experience using the technology [35,36,84,86,93,95]. Older adults were found to spend longer on websites than younger people [64,72] and were more likely to engage in data entry [64]. Two initiatives reported engagement with the technology and found reduced participant engagement over time [63,83].

#### Usability and Boundaries

Videoconference was deemed appropriate for educational sessions and other talking-based therapies [20] and to assess visually striking conditions (eg, wounds, ulcers, and edema) [88]. It was less useful when a hands-on approach is needed, such as for oral preventive care [30,31], physiotherapy or other active rehabilitative procedures [20,83], and for selected health conditions (eg, pneumonia) [35]. Clinicians have highlighted difficulties using videoconference when patients exhibited significant cognitive, sensory, and physical impairment [16,17,19,41,42]. Patients did not find some virtual educational or support interventions useful if they were already familiar with their conditions or if they had a straightforward recovery process (for post-discharge interventions) [65,84,110].

### Access for Individual Participants

A stable technology platform and appropriate physical environment were critical for telemonitoring [80,81,87]. Adherence among older people was facilitated by rapid feedback and access to providers when needed and the availability and clarity of protocols for missed readings or data entry [96]. For web-based initiatives, the key enablers for older adults were previous internet self-efficacy and, when compared with the younger population, higher leisure time to interact with web features and willingness to invest time in health [64]. For providers, flexible as well as appropriate funding and reimbursement were crucial [39,80,81].

### Limitations for Delivery at Scale

Ten studies reported individual access issues. The reasons included poor internet connectivity or speed (particularly in rural areas; n=4) [19,20,39,65] or equipment issues (n=4) [45,85,88,99], user error [88], and other technical problems (n=3) [20,24-26,39]. For older people, a lack of digital literacy also contributes to reduced motivation to access virtual care (n=2) [64,65].

From the providers’ perspective, a key challenge was staffing issues (n=6), including insufficient staff to run the modality [17,19,39], and a need for additional support owing to low staff digital literacy and change in common practice [35,83]. Another challenge included a lack of motivation among providers to use new technology (n=5) [17,20,33,39,94]. Management and relationship challenges were noted in residential aged care settings (n=3), driven by poor infrastructure, short project turnaround time, and high turnover of staff [35,39,116]. There were reports of complex mandates at various levels of government [35,94] and frustration with virtual care policies [39].

### Other WHO Digital Health Framework Items

A brief synthesis of technology and platform, adaptability, interoperability, replicability, data security, and regulatory compliance is provided in Multimedia Appendix 1. In total, 17 initiatives reported integrating virtual care into existing infrastructure and systems. No studies have reported issues regarding interoperability. However, this does not mean interoperability with existing systems was not an issue but that integration is often outside the capability and capacity of the research and operational teams. Most initiatives were funded by federal agencies (n=52) or state agencies (n=16), and a small number were funded by commercial or nonprofit organizations (Multimedia Appendix 1).

## Discussion

### Overview of Evidence of Virtual Care Use for Older Australians

This scoping review identified a wide range of virtual care modalities used for diverse care purposes and disease domains in older patients that have been tested or implemented in Australia. Across the 80 identified initiatives, older Australians were highly accepting of virtual care, in agreement with a recent survey [117]. Older Australians reported improved access to care, time efficiency, and self-management capacity in alignment with reviews of other modality- or disease-specific virtual care [118,119]. It remains challenging to define the exact use cases for the different virtual care modalities because of the variations in measured health or behavioral outcomes, patient conditions, frequency of use, and others. However, videoconference appears to be appropriate for most talking-based therapies and diagnosing visually evident conditions [20,88] and inappropriate for care needing hands-on approaches [20,30,31,35,83]. Telemonitoring or device use are appropriate options for interventions intended for self-management and monitoring, particularly for older adults with chronic diseases [84,86,96,115]. Web-based interventions and apps are convenient modalities for asynchronous delivery of information or educational interventions provided older people-friendly features are present (eg, large fonts) [64,72,76,99]. The findings of telephone interventions were most inconsistent, but the modality is widely used for follow-up calls and health coaching. Importantly, most studies we reviewed suggest that when delivered as a stand-alone intervention, the virtual care delivery model may yield comparable outcomes to in-person care when care needs and modality are aligned.

### Practical Considerations of Virtually Enabled Care for Older Adults

**Figure 2:**
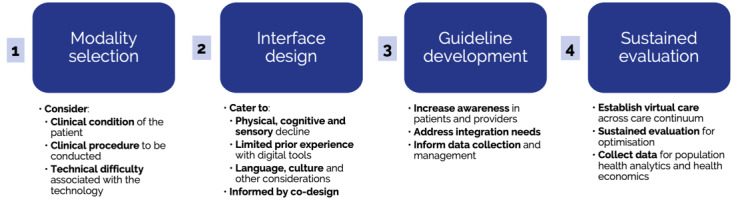
Key recommendations from this review regarding modality selection, interface design, guideline development, and sustained evaluation of virtual care initiatives in the older adult population.

Clinical indications for the use and boundaries of various virtual care modalities for older Australians generally echoed studies from other countries [120] and of the general population [121]. However, for older people, interface design should be user-friendly [16,24-26,28,76] and must cater to potential cognitive, sensory, and physical impairments [16,17,19,41,42]. The reduced engagement of older Australians over time should also be anticipated across modalities [63,83], as has been identified globally [122]. Reasons are poorly reported; however, this may be attributable to high effectiveness (leading to early disengagement), as reported in a US study [123], or a lack thereof [48,98,100,103,105,107,109-111]. Altogether, these findings suggest the importance of engaging older adults across all stages of initiative development (ie, using a co-design approach; Figure 2). A growing commercial interest in digital health in Australia may also lead to a wider variety of options for equipment and technology in the near future [124].

### Older Australians Are Ready to Be Digitally Equipped and Use Virtual Care

Many older adults want to sustain their independence and self-manage their health [7]. This may explain the indications for higher engagement in older versus young people [64,72,73]. While lack of technical literacy in older people remains an issue globally [118,119,125] and in Australia [64,65], this is likely modifiable. For example, studies in our review [35,36,84,86,93,95,96] and in another similar review [126] suggest that equipment training and clear protocols for independent activities (eg, data entry) may help reduce anxiety and negative perceptions toward new technology and improve adherence (Figure 2). Internet literacy rates among older Australians have also improved (6% in 2001 and 79% in 2015) [127], with a survey in 2018 indicating “unnecessity” (80%) as the reason for no internet use in the last 3 months versus 20% for “no confidence/knowledge” [128]. Therefore, while the digital divide still exists among older Australians, advanced age is unlikely to be the main barrier to virtual care use [45,93].

### While Older Adults Are Ready for Virtual Care, Challenges Faced by Providers Remain

Staffing, bureaucracy, and management issues were identified as challenges by Australian providers and are echoed globally [129,130]. These barriers have been reported together with a lack of motivation among providers [17,39], suggesting that they go hand in hand. Interestingly, low digital literacy among staff has been observed, leading to the need for recurrent training [35]. Indeed, digital literacy criteria are not an integral part of staff recruitment for Australian care providers [131], highlighting the importance of implementation of digital health education strategy [132]. Furthermore, only a quarter of all initiatives evaluated a cost-related component, wherein recurring staff training and logistics were large contributors to capital costs [23,30,31,45,50,59,84,93]. More cost-related data are needed to robustly inform decision-making, including for scale-up considerations.

### There Is a Need for Digital Health Policy Surrounding Virtual Care for Older People

Most identified studies were early-stage (pilot) evaluations, highlighting the need for a larger body of evidence from sustained and integrated implementation trials. We also found limited cost-related data or economic evaluations, which are important parameters to inform wider adoption of virtual care services. Therefore, institutions and care providers may benefit from the provision of set standards or guidelines for virtually enabled care of older people. Such guidelines currently do not exist in Australia. The Aged Care Industry Information Technology Council report, which summarized technological innovations across the globe, as well as key learnings from this review, may serve as starting points [133]. There should also be strong recommendations for the collection and evaluation of critical data (eg, clinical, legislative, and economic data) to appropriately inform, fund, and mobilize virtual care services. The key recommendations are summarized in Figure 2.

### Strengths and Limitations

The strength of this review is that it brings together the evidence of the broad range of virtual care modalities tested to support older adults in managing their chronic health conditions. These findings are also likely relevant for other countries with a similar demographic profile (ie, aging populations and high-income countries) or technological aptitude among their older populations [134] and for countries at a similar stage of digital health adoption [135].

The limitations of this study are as follows: to ensure a wide coverage of references and timely identification of evidence, we only included articles from the past 10 years. In terms of the evidence pooled, we included studies with varying definitions of older adults; thus, generalizations may not apply to all older adult populations (eg, people in their 70s may have very different well-being and technological characteristics compared with those in their 50s or 60s) [136]. There is also a large heterogeneity across studies and outcomes reported in this scoping review, which makes it challenging to draw sweeping conclusions about one modality or population against another. A network meta-analysis may be a suitable next step to examine all the comparisons for different elements that could be included in virtual care interventions and control conditions. Finally, conclusions drawn from randomized controlled trials in the context of virtual care are often subject to publication bias. Nevertheless, this review provides critical first steps to develop a virtual care policy for older people, particularly in terms of key elements for consideration of surrounding modality selection, interface considerations, and need for guideline development and sustained evaluations.

### Conclusions

This review identified that there are a wide range of virtual care modalities designed to enable older adults to manage their chronic health conditions. The identified barriers to wider adoption were attributable to physical, cognitive, or sensory impairment at the patient level and staffing, legislative, and motivational issues among providers at the system level. More evidence from embedded and integrative evaluations are needed to ensure virtually enabled care can be used more widely and efficiently by providers and older Australians.

## Appendix

Multimedia Appendix 1 Search strategy, inclusion criteria, PRISMA flowchart, WHO Digital Health framework, and additional data synthesis.

## Abbreviations

ICER: incremental cost-effectiveness ratio
PRISMA: Preferred Reporting Items for Systematic Reviews and Meta-Analyses
PRISMA-ScR: Preferred Reporting Items for Systematic Reviews and Meta-Analyses extension for Scoping Reviews
WHO: World Health Organization

## References

[1] (2019). The Australian health system. Australian Government Department of Health.

[2] Dougherty S, Lorenzoni L, Marino A, Murtin F (2022). The impact of decentralisation on the performance of health care systems: a non-linear relationship. Eur J Health Econ.

[3] Anthony Jr B (2020). Use of telemedicine and virtual care for remote treatment in response to COVID-19 pandemic. J Med Syst.

[4] Angus D, Connolly M, Salita M, Firor P (2020). The shift to virtual care in response to COVID-19. PwC Australia.

[5] (2020). Digital health. Australian Institute of Health and Welfare.

[6] (2021). Older Australians - Health—service use. Australian Institute of Health and Welfare.

[7] Abdi S, Spann A, Borilovic J, de Witte L, Hawley M (2019). Understanding the care and support needs of older people: a scoping review and categorisation using the WHO international classification of functioning, disability and health framework (ICF). BMC Geriatr.

[8] (2021). Aged Care. Australian Institute of Health and Welfare.

[9] (2016). 4430.0 - Disability, Ageing and Carers, Australia: Summary of Findings, 2015 - Older People. Australian Bureau of Statistics.

[10] Thomas J, Barraket J, Wilson CK, Holcombe-James I, Kennedy J, Rennie E, Ewing S, MacDonald T (2020). Measuring Australia’s Digital Divide: The Australian Digital Inclusion Index 2020.

[11] Grant MJ, Booth A (2009). A typology of reviews: an analysis of 14 review types and associated methodologies. Health Info Libr J.

[12] Tricco AC, Lillie E, Zarin W, O'Brien KK, Colquhoun H, Levac D, Moher D, Peters MD, Horsley T, Weeks L, Hempel S, Akl EA, Chang C, McGowan J, Stewart L, Hartling L, Aldcroft A, Wilson MG, Garritty C, Lewin S, Godfrey CM, Macdonald MT, Langlois EV, Soares-Weiser K, Moriarty J, Clifford T, Tunçalp Ö, Straus SE (2018). PRISMA extension for scoping reviews (PRISMA-ScR): checklist and explanation. Ann Intern Med.

[13] (2016). Monitoring and evaluating digital health interventions: a practical guide to conducting research and assessment. World Health Organization.

[14] Scott IA (2009). Non-inferiority trials: determining whether alternative treatments are good enough. Med J Aust.

[15] Banbury A, Chamberlain D, Nancarrow S, Dart J, Gray L, Parkinson L (2017). Can videoconferencing affect older people's engagement and perception of their social support in long-term conditions management: a social network analysis from the Telehealth Literacy Project. Health Soc Care Community.

[16] Dham P, Gupta N, Alexander J, Black W, Rajji T, Skinner E (2018). Community based telepsychiatry service for older adults residing in a rural and remote region- utilization pattern and satisfaction among stakeholders. BMC Psychiatry.

[17] Moyle W, Jones C, Murfield J, Liu F (2020). 'For me at 90, it's going to be difficult': feasibility of using iPad video-conferencing with older adults in long-term aged care. Aging Ment Health.

[18] Martin-Khan M, Flicker L, Wootton R, Loh PK, Edwards H, Varghese P, Byrne GJ, Klein K, Gray LC (2012). The diagnostic accuracy of telegeriatrics for the diagnosis of dementia via video conferencing. J Am Med Dir Assoc.

[19] Venuthurupalli SK, Rolfe A, Fanning J, Cameron A, Hoy WE, NHMRC CKD.CRE and the CKD.QLD Collaborative (2018). Chronic Kidney Disease, Queensland (CKD.QLD) registry: management of CKD With telenephrology. Kidney Int Rep.

[20] Wundersitz C, Caelli A, Georgy J, Musovic A, Manning R, Prause M, Robertson J, Taylor NF (2020). Conducting community rehabilitation review sessions via videoconference: a feasibility study. Aust J Rural Health.

[21] Bladin CF, Moloczij N, Ermel S, Bagot KL, Kilkenny M, Vu M, Cadilhac DA, VST program investigators (2015). Victorian Stroke Telemedicine Project: implementation of a new model of translational stroke care for Australia. Intern Med J.

[22] Bladin CF, Kim J, Bagot KL, Vu M, Moloczij N, Denisenko S, Price C, Pompeani N, Arthurson L, Hair C, Rabl J, O'Shea M, Groot P, Bolitho L, Campbell BC, Dewey HM, Donnan GA, Cadilhac DA (2020). Improving acute stroke care in regional hospitals: clinical evaluation of the Victorian Stroke Telemedicine program. Med J Aust.

[23] Nagao KJ, Koschel A, Haines HM, Bolitho LE, Yan B (2012). Rural Victorian telestroke project. Intern Med J.

[24] Burns CL, Ward EC, Hill AJ, Malcolm K, Bassett L, Kenny LM, Greenup P (2012). A pilot trial of a speech pathology telehealth service for head and neck cancer patients. J Telemed Telecare.

[25] Burns CL, Ward EC, Hill AJ, Kularatna S, Byrnes J, Kenny LM (2017). Randomized controlled trial of a multisite speech pathology telepractice service providing swallowing and communication intervention to patients with head and neck cancer: evaluation of service outcomes. Head Neck.

[26] Burns CL, Kularatna S, Ward EC, Hill AJ, Byrnes J, Kenny LM (2017). Cost analysis of a speech pathology synchronous telepractice service for patients with head and neck cancer. Head Neck.

[27] Host BK, Turner AW, Muir J (2018). Real-time teleophthalmology video consultation: an analysis of patient satisfaction in rural Western Australia. Clin Exp Optom.

[28] Jiang B, Bills M, Poon P (2023). Integrated telehealth-assisted home-based specialist palliative care in rural Australia: a feasibility study. J Telemed Telecare.

[29] Lillicrap T, Pinheiro A, Miteff F, Garcia-Bermejo P, Gangadharan S, Wellings T, O'Brien B, Evans J, Alanati K, Bivard A, Parsons M, Levi C, Garcia-Esperon C, Spratt N (2020). No evidence of the "weekend effect" in the Northern New South Wales telestroke network. Front Neurol.

[30] Mariño R, Tonmukayakul U, Marwaha P, Collmann R, Hopcraft M, Manton DJ, Stranieri A, Clarke K (2014). Teleconsultation/telediagnosis using teledentistry technology: a pilot feasibility study. Int J Adv Life Sci.

[31] Mariño R, Tonmukayakul U, Manton D, Stranieri A, Clarke K (2016). Cost-analysis of teledentistry in residential aged care facilities. J Telemed Telecare.

[32] Sabesan S, Larkins S, Evans R, Varma S, Andrews A, Beuttner P, Brennan S, Young M (2012). Telemedicine for rural cancer care in North Queensland: bringing cancer care home. Aust J Rural Health.

[33] Stillerova T, Liddle J, Gustafsson L, Lamont R, Silburn P (2016). Could everyday technology improve access to assessments? A pilot study on the feasibility of screening cognition in people with Parkinson's disease using the Montreal Cognitive Assessment via Internet videoconferencing. Aust Occup Ther J.

[34] Tam A, Leung A, O'Callaghan C, Fagermo N (2017). Role of telehealth in perioperative medicine for regional and rural patients in Queensland. Intern Med J.

[35] Wade V, Whittaker F, Hamlyn J (2015). An evaluation of the benefits and challenges of video consulting between general practitioners and residential aged care facilities. J Telemed Telecare.

[36] Towers C, Tyler M (2014). The broadband-enabled innovation program: a working demonstration of the effective use of technology in community-based patient care. Aust Fam Physician.

[37] Taylor A, Morris G, Pech J, Rechter S, Carati C, Kidd MR (2015). Home telehealth video conferencing: perceptions and performance. JMIR Mhealth Uhealth.

[38] Theodoros DG, Hill AJ, Russell TG (2016). Clinical and quality of life outcomes of speech treatment for Parkinson's disease delivered to the home via telerehabilitation: a noninferiority randomized controlled trial. Am J Speech Lang Pathol.

[39] Jones N (2017). Final report of the Better Health Care Connections Video Consultation Project in the Frankston and Mornington Peninsula region. South Eastern Melbourne Primary Health Network.

[40] Dorsey K (2017). GP video consultations a success in residential aged care. Partyline.

[41] Ward EC, Burns CL, Theodoros DG, Russell TG (2014). Impact of dysphagia severity on clinical decision making via telerehabilitation. Telemed J E Health.

[42] Ward EC, Sharma S, Burns C, Theodoros D, Russell T (2012). Validity of conducting clinical dysphagia assessments for patients with normal to mild cognitive impairment via telerehabilitation. Dysphagia.

[43] Hwang R, Bruning J, Morris NR, Mandrusiak A, Russell T (2017). Home-based telerehabilitation is not inferior to a centre-based program in patients with chronic heart failure: a randomised trial. J Physiother.

[44] Hwang R, Morris NR, Mandrusiak A, Bruning J, Peters R, Korczyk D, Russell T (2019). Cost-utility analysis of home-based telerehabilitation compared with centre-based rehabilitation in patients with heart failure. Heart Lung Circ.

[45] Katalinic O, Young A, Doolan D (2013). Case study: the interact home telehealth project. J Telemed Telecare.

[46] Beauchamp A, Mohebbi M, Cooper A, Pridmore V, Livingston P, Scanlon M, Davis M, O'Hara J, Osborne R (2020). The impact of translated reminder letters and phone calls on mammography screening booking rates: two randomised controlled trials. PLoS One.

[47] Almeida OP, Patel H, Kelly R, Ford A, Flicker L, Robinson S, Araya R, Gilbody S, Thompson S (2021). Preventing depression among older people living in rural areas: a randomised controlled trial of behavioural activation in collaborative care. Int J Geriatr Psychiatry.

[48] Cameron-Tucker HL, Wood-Baker R, Joseph L, Walters JA, Schüz N, Walters EH (2016). A randomized controlled trial of telephone-mentoring with home-based walking preceding rehabilitation in COPD. Int J Chron Obstruct Pulmon Dis.

[49] Lahham A, McDonald CF, Moore R, Cox NS, Rawlings S, Nichols A, Liacos A, Holland AE (2020). The impact of home-based pulmonary rehabilitation on people with mild chronic obstructive pulmonary disease: a randomised controlled trial. Clin Respir J.

[50] Lannin NA, Anderson C, Lim J, Paice K, Price C, Faux S, Levi C, Donnan G, Cadilhac D (2013). Telephone follow-up was more expensive but more efficient than postal in a national stroke registry. J Clin Epidemiol.

[51] Hullick CJ, Hall AE, Conway JF, Hewitt JM, Darcy LF, Barker RT, Oldmeadow C, Attia JR (2021). Reducing hospital transfers from aged care facilities: a large-scale stepped wedge evaluation. J Am Geriatr Soc.

[52] Ling R, Searles A, Hewitt J, Considine R, Turner C, Thomas S, Thomas K, Drinkwater K, Higgins I, Best K, Conway J, Hullick C (2019). Cost analysis of an integrated aged care program for residential aged care facilities. Aust Health Rev.

[53] Doyle C, Bhar S, Fearn M, Ames D, Osborne D, You E, Gorelik A, Dunt D (2017). The impact of telephone-delivered cognitive behaviour therapy and befriending on mood disorders in people with chronic obstructive pulmonary disease: a randomized controlled trial. Br J Health Psychol.

[54] Moayeri F, Dunt D, Hsueh YS, Doyle C (2019). Cost-utility analysis of telephone-based cognitive behavior therapy in chronic obstructive pulmonary disease (COPD) patients with anxiety and depression comorbidities: an application for willingness to accept concept. Expert Rev Pharmacoecon Outcomes Res.

[55] Price P, Tacey M, Koufariotis V, Stramandinoli D, Vincent R, Grigg L, Zentner D (2018). A contemporary phone-based cardiac coaching program: evolution and cross cultural utility. Heart Lung Circ.

[56] Regan AK, Bloomfield L, Peters I, Effler PV (2017). Randomized controlled trial of text message reminders for increasing influenza vaccination. Ann Fam Med.

[57] Sampurno F, Ruseckaite R, Millar JL, Evans SM (2016). Comparison of patient-reported quality-of-life and complications in men with prostate cancer, between two modes of administration. Clin Genitourin Cancer.

[58] Tang D, Mitchell P, Liew G, Burlutsky G, Flood VM, Gopinath B (2020). Telephone-delivered dietary intervention in patients with age-related macular degeneration: 3-month post-intervention findings of a randomised controlled trial. Nutrients.

[59] Tutty E, Petelin L, McKinley J, Young MA, Meiser B, Rasmussen VM, Forbes Shepherd R, James PA, Forrest LE (2019). Evaluation of telephone genetic counselling to facilitate germline BRCA1/2 testing in women with high-grade serous ovarian cancer. Eur J Hum Genet.

[60] Voukelatos A, Merom D, Sherrington C, Rissel C, Cumming RG, Lord SR (2015). The impact of a home-based walking programme on falls in older people: the Easy Steps randomised controlled trial. Age Ageing.

[61] Walters J, Cameron-Tucker H, Wills K, Schüz N, Scott J, Robinson A, Nelson M, Turner P, Wood-Baker R, Walters EH (2013). Effects of telephone health mentoring in community-recruited chronic obstructive pulmonary disease on self-management capacity, quality of life and psychological morbidity: a randomised controlled trial. BMJ Open.

[62] Young AM, Mudge AM, Banks MD, Rogers L, Demedio K, Isenring E (2018). Improving nutritional discharge planning and follow up in older medical inpatients: hospital to home outreach for malnourished elders. Nutr Diet.

[63] Hammersley ML, Cann VR, Parrish AM, Jones RA, Holloway DJ (2015). Evaluation of the effects of a telephone-delivered health behaviour change program on weight and physical activity. Nutr Diet.

[64] Alley SJ, Kolt GS, Duncan MJ, Caperchione CM, Savage TN, Maeder AJ, Rosenkranz RR, Tague R, Van Itallie AK, Kerry Mummery W, Vandelanotte C (2018). The effectiveness of a web 2.0 physical activity intervention in older adults - a randomised controlled trial. Int J Behav Nutr Phys Act.

[65] Burns P, Jones SC, Iverson D, Caputi P (2013). AsthmaWise - a field of dreams? The results of an online education program targeting older adults with asthma. J Asthma.

[66] Kiropoulos LA, Griffiths KM, Blashki G (2011). Effects of a multilingual information website intervention on the levels of depression literacy and depression-related stigma in Greek-born and Italian-born immigrants living in Australia: a randomized controlled trial. J Med Internet Res.

[67] O'moore KA, Newby JM, Andrews G, Hunter DJ, Bennell K, Smith J, Williams AD (2018). Internet cognitive-behavioral therapy for depression in older adults with knee osteoarthritis: a randomized controlled trial. Arthritis Care Res (Hoboken).

[68] Staples LG, Fogliati VJ, Dear BF, Nielssen O, Titov N (2016). Internet-delivered treatment for older adults with anxiety and depression: implementation of the Wellbeing Plus Course in routine clinical care and comparison with research trial outcomes. BJPsych Open.

[69] Titov N, Fogliati VJ, Staples LG, Gandy M, Johnston L, Wootton B, Nielssen O, Dear BF (2016). Treating anxiety and depression in older adults: randomised controlled trial comparing guided v. self-guided Internet-delivered cognitive-behavioural therapy. BJPsych Open.

[70] Titov N, Dear BF, Ali S, Zou JB, Lorian CN, Johnston L, Terides MD, Kayrouz R, Klein B, Gandy M, Fogliati VJ (2015). Clinical and cost-effectiveness of therapist-guided internet-delivered cognitive behavior therapy for older adults with symptoms of depression: a randomized controlled trial. Behav Ther.

[71] Torrens E, Walker SM (2017). Demographic characteristics of Australian health consumers who were early registrants for opt-in personally controlled electronic health records. Health Inf Manag.

[72] Vandelanotte C, Ammann RA, De Vries H, Mummery K (2012). Can a website-delivered computer-tailored physical activity intervention be acceptable, usable, and effective for older people?. J Sci Med Sport.

[73] Wilson CJ, Flight IH, Turnbull D, Gregory T, Cole SR, Young GP, Zajac IT (2015). A randomised controlled trial of personalised decision support delivered via the internet for bowel cancer screening with a faecal occult blood test: the effects of tailoring of messages according to social cognitive variables on participation. BMC Med Inform Decis Mak.

[74] Staffieri SE, Ruddle JB, Kearns LS, Barbour JM, Edwards TL, Paul P, Mackey DA (2011). Telemedicine model to prevent blindness from familial glaucoma. Clin Exp Ophthalmol.

[75] Cadilhac DA, Andrew NE, Busingye D, Cameron J, Thrift AG, Purvis T, Li JC, Kneebone I, Thijs V, Hackett ML, Lannin NA, Kilkenny MF, ReCAPS investigators (2020). Pilot randomised clinical trial of an eHealth, self-management support intervention (iVERVE) for stroke: feasibility assessment in survivors 12-24 months post-event. Pilot Feasibility Stud.

[76] Bhattarai P, Newton-John TR, Phillips JL (2020). Apps for pain self-management of older people's arthritic pain, one size doesn't fit all: a qualitative study. Arch Gerontol Geriatr.

[77] Thomas R, Michaleff ZA, Greenwood H, Abukmail E, Glasziou P (2020). Concerns and misconceptions about the Australian government's COVIDSafe app: cross-sectional survey study. JMIR Public Health Surveill.

[78] Tongpeth J, Du H, Barry T, Clark RA (2020). Effectiveness of an Avatar application for teaching heart attack recognition and response: a pragmatic randomized control trial. J Adv Nurs.

[79] Wonggom P, Nolan P, Clark RA, Barry T, Burdeniuk C, Nesbitt K, O'Toole K, Du H (2020). Effectiveness of an avatar educational application for improving heart failure patients' knowledge and self-care behaviors: a pragmatic randomized controlled trial. J Adv Nurs.

[80] Nancarrow S, Roots A, Banbury A, Barlo K (2014). Feros Care's My Health Clinic at Home pilot: Final report. Feros Care & Southern Cross University.

[81] (2014). Feros Care's My Health Clinic At Home Pilot: Summary Report. Feros Care & Southern Cross University.

[82] Nancarrow S, Banbury A, Buckley J (2016). Evaluation of a National Broadband Network-enabled Telehealth trial for older people with chronic disease. Aust Health Rev.

[83] Tieman JJ, Swetenham K, Morgan DD, To TH, Currow DC (2016). Using telehealth to support end of life care in the community: a feasibility study. BMC Palliat Care.

[84] De San Miguel K, Smith J, Lewin G (2013). Telehealth remote monitoring for community-dwelling older adults with chronic obstructive pulmonary disease. Telemed J E Health.

[85] Ding H, Jayasena R, Chen SH, Maiorana A, Dowling A, Layland J, Good N, Karunanithi M, Edwards I (2020). The effects of telemonitoring on patient compliance with self-management recommendations and outcomes of the innovative telemonitoring enhanced care program for chronic heart failure: randomized controlled trial. J Med Internet Res.

[86] Bereznicki LR, Jackson SL, Peterson GM (2013). Supervised patient self-testing of warfarin therapy using an online system. J Med Internet Res.

[87] Karunanithi M, Zhang Q (2018). An innovative technology to support independent living: the smarter safer homes platform. Stud Health Technol Inform.

[88] Wade R, Shaw K, Cartwright C (2012). Factors affecting provision of successful monitoring in home Telehealth. Gerontology.

[89] Schoene D, Valenzuela T, Toson B, Delbaere K, Severino C, Garcia J, Davies TA, Russell F, Smith ST, Lord SR (2015). Interactive cognitive-motor step training improves cognitive risk factors of falling in older adults - a randomized controlled trial. PLoS One.

[90] Schoene D, Lord SR, Verhoef P, Smith ST (2011). A novel video game--based device for measuring stepping performance and fall risk in older people. Arch Phys Med Rehabil.

[91] Schoene D, Lord SR, Delbaere K, Severino C, Davies TA, Smith ST (2013). A randomized controlled pilot study of home-based step training in older people using videogame technology. PLoS One.

[92] Schoene D, Smith ST, Davies TA, Delbaere K, Lord SR (2014). A Stroop Stepping Test (SST) using low-cost computer game technology discriminates between older fallers and non-fallers. Age Ageing.

[93] (2017). Evaluation of the In-Home Telemonitoring for Veterans trial. Health Outcomes International.

[94] Celler B, Varnfield M, Sparks R, Li J, Nepal S, Jang-Jaccard J, McBride S, Jayasena R (2016). Home monitoring of chronic disease for aged care. Australian e-Health Research Centre, CSIRO.

[95] Halcomb E, Purcell R, Hickman L, Smyth E (2016). Telemonitoring is acceptable amongst community dwelling older Australians with chronic conditions. Collegian.

[96] Chow JS, Gonzalez-Arce V, Knight A, Kohler F (2018). Retrospective analysis of telemonitoring in Wollondilly, Australia. J Integr Care.

[97] Pasalich M, Lee AH, Jancey J, Burke L, Howat P (2013). Sustainability of a physical activity and nutrition program for seniors. J Nutr Health Aging.

[98] Wootton SL, Hill K, Alison JA, Ng LW, Jenkins S, Eastwood PR, Hillman DR, Jenkins C, Spencer LM, Cecins N, McKeough ZJ (2019). Effects of ongoing feedback during a 12-month maintenance walking program on daily physical activity in people with COPD. Lung.

[99] Haynes A, Sherrington C, Wallbank G, Lester D, Tong A, Merom D, Rissel C, Tiedemann A (2021). "Someone's got my back": older people's experience of the coaching for healthy ageing program for promoting physical activity and preventing falls. J Aging Phys Act.

[100] Brickwood KJ, Ahuja KD, Watson G, O'Brien JA, Williams AD (2021). Effects of activity tracker use with health professional support or telephone counseling on maintenance of physical activity and health outcomes in older adults: randomized controlled trial. JMIR Mhealth Uhealth.

[101] Jancey JM, Lee AH, Howat PA, Burke L, Leong CC, Shilton T (2011). The effectiveness of a walking booster program for seniors. Am J Health Promot.

[102] Menant JC, Migliaccio AA, Sturnieks DL, Hicks C, Lo J, Ratanapongleka M, Turner J, Delbaere K, Titov N, Meinrath D, McVeigh C, Close JC, Lord SR (2018). Reducing the burden of dizziness in middle-aged and older people: a multifactorial, tailored, single-blind randomized controlled trial. PLoS Med.

[103] Williams A, Manias E, Walker R, Gorelik A (2012). A multifactorial intervention to improve blood pressure control in co-existing diabetes and kidney disease: a feasibility randomized controlled trial. J Adv Nurs.

[104] Padayachee A, Ranatunga C, Comans TA (2019). Utilising capacity in a rural hospital to support older people requiring hospital care: Kilcoy Connect. Aust J Rural Health.

[105] Gallagher C, Orchard J, Nyfort-Hansen K, Sanders P, Neubeck L, Hendriks JM (2020). NursE led atrial fibrillation management: the NEAT study: a randomized controlled trial. J Cardiovasc Nurs.

[106] Clemson L, Lannin NA, Wales K, Salkeld G, Rubenstein L, Gitlin L, Barris S, Mackenzie L, Cameron ID (2016). Occupational therapy predischarge home visits in acute hospital care: a randomized trial. J Am Geriatr Soc.

[107] Provencher V, Clemson L, Wales K, Cameron ID, Gitlin LN, Grenier A, Lannin NA (2020). Supporting at-risk older adults transitioning from hospital to home: who benefits from an evidence-based patient-centered discharge planning intervention? Post-hoc analysis from a randomized trial. BMC Geriatr.

[108] Wales K, Salkeld G, Clemson L, Lannin NA, Gitlin L, Rubenstein L, Howard K, Howell M, Cameron ID (2018). A trial based economic evaluation of occupational therapy discharge planning for older adults: the HOME randomized trial. Clin Rehabil.

[109] Sharma Y, Thompson CH, Kaambwa B, Shahi R, Hakendorf P, Miller M (2017). Investigation of the benefits of early malnutrition screening with telehealth follow up in elderly acute medical admissions. QJM.

[110] Young JM, Butow PN, Walsh J, Durcinoska I, Dobbins TA, Rodwell L, Harrison JD, White K, Gilmore A, Hodge B, Hicks H, Smith S, O'Connor G, Byrne CM, Meagher AP, Jancewicz S, Sutherland A, Ctercteko G, Pathma-Nathan N, Curtin A, Townend D, Abraham NS, Longfield G, Rangiah D, Young CJ, Eyers A, Lee P, Fisher D, Solomon MJ (2013). Multicenter randomized trial of centralized nurse-led telephone-based care coordination to improve outcomes after surgical resection for colorectal cancer: the CONNECT intervention. J Clin Oncol.

[111] Harrison JD, Young JM, Solomon MJ, Butow PN, Secomb R, Masya L (2011). Randomized pilot evaluation of the supportive care intervention "CONNECT" for people following surgery for colorectal cancer. Dis Colon Rectum.

[112] Walters JA, Cameron-Tucker H, Courtney-Pratt H, Nelson M, Robinson A, Scott J, Turner P, Walters EH, Wood-Baker R (2012). Supporting health behaviour change in chronic obstructive pulmonary disease with telephone health-mentoring: insights from a qualitative study. BMC Fam Pract.

[113] White VM, Macvean ML, Grogan S, D'Este C, Akkerman D, Ieropoli S, Hill DJ, Sanson-Fisher R (2012). Can a tailored telephone intervention delivered by volunteers reduce the supportive care needs, anxiety and depression of people with colorectal cancer? A randomised controlled trial. Psychooncology.

[114] Suttanon P, Hill KD, Said CM, Williams SB, Byrne KN, LoGiudice D, Lautenschlager NT, Dodd KJ (2013). Feasibility, safety and preliminary evidence of the effectiveness of a home-based exercise programme for older people with Alzheimer's disease: a pilot randomized controlled trial. Clin Rehabil.

[115] Church A (2019). Virtual Clinical Care Home Telemonitoring Service: A hospital avoidance strategy in regional South Australia. SA Health.

[116] Masso MR, Samsa PD, Fildes DL, Duncan C (2015). Evaluation of the Better Health Care Connections: Models for Short Term, More Intensive Health Care for Aged Care Recipients Program final report. Australian Health Services Research Institute.

[117] (2020). Telehealth - here to stay? Key insights from an expansive study into Australia’s response to COVID-19. Global Centre for Modern Ageing.

[118] Kruse C, Fohn J, Wilson N, Nunez Patlan E, Zipp S, Mileski M (2020). Utilization barriers and medical outcomes commensurate with the use of telehealth among older adults: systematic review. JMIR Med Inform.

[119] Doraiswamy S, Jithesh A, Mamtani R, Abraham A, Cheema S (2021). Telehealth use in geriatrics care during the COVID-19 pandemic-a scoping review and evidence synthesis. Int J Environ Res Public Health.

[120] Vergara J, Parish A, Smallheer B (2020). Telehealth: opportunities in geriatric patient care during COVID-19. Geriatr Nurs.

[121] Sirintrapun SJ, Lopez AM (2018). Telemedicine in cancer care. Am Soc Clin Oncol Educ Book.

[122] Gorst SL, Armitage CJ, Brownsell S, Hawley MS (2014). Home telehealth uptake and continued use among heart failure and chronic obstructive pulmonary disease patients: a systematic review. Ann Behav Med.

[123] Chien I, Enrique A, Palacios J, Regan T, Keegan D, Carter D, Tschiatschek S, Nori A, Thieme A, Richards D, Doherty G, Belgrave D (2020). A machine learning approach to understanding patterns of engagement with Internet-delivered mental health interventions. JAMA Netw Open.

[124] Coote D (2021). Article: Why the Telehealth industry is poised for significant growth. BDO Auustralia.

[125] Foster MV, Sethares KA (2014). Facilitators and barriers to the adoption of telehealth in older adults: an integrative review. Comput Inform Nurs.

[126] Maeder A, Poultney N, Morgan G, Lippiatt R (2015). Patient compliance in home-based self-care telehealth projects. J Telemed Telecare.

[127] Wilding R, Malta S (2018). Digital divide: getting seniors online. La Trobe University.

[128] (2020). Use of information technology by people with disability, older people and primary carers. Australian Bureau of Statistics.

[129] Gajarawala SN, Pelkowski JN (2021). Telehealth benefits and barriers. J Nurse Pract.

[130] Almathami HK, Win KT, Vlahu-Gjorgievska E (2020). Barriers and facilitators that influence telemedicine-based, real-time, online consultation at patients' homes: systematic literature review. J Med Internet Res.

[131] (2021). Inspiring new models of care: Digital health in the home. Where are we now? Where to from here?. Global Centre for Modern Ageing.

[132] (2022). National Digital Health Workforce and Education Roadmap. Australian Digital Health Agency.

[133] (2019). Aged Care Industry Technology Council releases a report on what the Research and Evidence is indicating right now in the sector…. Aged Care Industry Information Technology Council.

[134] Lam K, Lu AD, Shi Y, Covinsky KE (2020). Assessing telemedicine unreadiness among older adults in the United States during the COVID-19 pandemic. JAMA Intern Med.

[135] Bhaskar S, Bradley S, Chattu VK, Adisesh A, Nurtazina A, Kyrykbayeva S, Sakhamuri S, Yaya S, Sunil T, Thomas P, Mucci V, Moguilner S, Israel-Korn S, Alacapa J, Mishra A, Pandya S, Schroeder S, Atreja A, Banach M, Ray D (2020). Telemedicine across the globe-position paper from the COVID-19 pandemic health system resilience PROGRAM (REPROGRAM) International Consortium (Part 1). Front Public Health.

[136] (2018). Understanding the digital behaviours of older Australians: Summary of national survey and qualitative research. Office of the eSafety Commissioner, Australian Government.

