# Supplementary material for: Virtual Care Initiatives for Older Adults in Australia: Scoping Review
Source: J Med Internet Res. 2023 Jan 18;25:e38081. doi: 10.2196/38081 (PMC9892987; doi:10.2196/38081)
Supplement: Multimedia Appendix 1 [file jmir_v25i1e38081_app1.docx]

**Supplementary Appendix**

**Supplementary Appendix 1 – Search details and sample search strategy**

Sources:

- Five health and multidisciplinary databases including Medline via Ovid, Embase via Ovid, Psycinfo via Ovid, CINAHL via EBSCOHost, and Ageline via EBSCOHost were searched.
- Google Advanced Search was used to locate grey literature documents in Analysis & Policy Observatory (site: apo.org.au), as well as Australian web domains: .gov.au, .org.au, .edu.au., .nsw.gov.au, .vic.gov.au, .wa.gov.au, .sa.gov.au, .nt.gov.au .tas.gov.au, and .qld.gov.au. For each of the search, the first 100 results were screened.
- Informit (search.informit.org) and International HTA Database (database.inahta.org) were also searched for additional grey literature documents.
- Manual handsearching on Google and government websites and reference screening.

Date of search: 8 March 2021

Database: Medline via Ovid

| **#** | **Searches** | **Results** | **Hedge and rationale** |
| --- | --- | --- | --- |
| 1 | (initiative* or educat* or health literacy or strateg* or test* or framework or implement* or interven* or integrat* or program* or innovat* or (health* adj deliver*) or (care adj deliver*) or reform* or redesign or guideline*).tw. | 7077934 | “Initiative” hedge – to locate specific initiatives which may include a broad or specific targeted health problem |
| 2 | Health Literacy/ or Health Education/ | 67431 |  |
| 3 | "delivery of health care"/ or "delivery of health care, integrated"/ or health care reform/ or health services accessibility/ or learning health system/ or healthcare disparities/ | 216790 |  |
| 4 | (virtual care or care virtual* or virtual health or digital* or digital* health or digital* care or digital storytell* or telemedicine or tele-medicine or telehealth or tele-health or telepractice or tele-practice or mobile health or mhealth or m-health or ehealth or e-health or emental or e-mental or smartphone* or (tablet adj computer) or phone* or telephone* or cellphone* or smartwatch* or (mobile adj phone) or online or web* or internet or remote).tw. | 642803 | “Virtual care” hedge – to capture various modalities of virtual care |
| 5 | exp telemedicine/ or exp remote consultation/ or exp telepathology/ or exp teleradiology/ or exp telerehabilitation/ | 32963 |  |
| 6 | exp Mobile Applications/ or exp Internet-Based Intervention/ | 7485 |  |
| 7 | (elder* or old-age or old* people or old* individual* or old* population or aged care or ageing or aging or old* patient* or older adult* or older person* or very old or geriatric or nursing home* or long term care or longterm care or residential aged care or LTCF or aged care facilit* or skilled nursing facilit* or institutionalised elder* or institutionalized elder*).tw. | 687839 | “Older adult” hedge – to narrow down search for older adults only |
| 8 | exp "Aged, 80 and over"/ or exp Health Services for the Aged/ or exp Homes for the Aged/ or exp Aged/ or exp Nursing Homes/ | 3229716 |  |
| 9 | Aging/ | 233307 |  |
| 10 | (australia* or new south wales or northern territory or victoria* or queensland* or tasmania*).mp. | 217462 | “Australia” hedge – to narrow down search to all of Australia and specific Australian states |
| 11 | australia/ or australian capital territory/ or new south wales/ or northern territory/ or queensland/ or south australia/ or tasmania/ or victoria/ or western australia/ | 149274 |  |
| 12 | 1 or 2 or 3 | 7212739 | All of “initiative” |
| 13 | 4 or 5 or 6 | 654178 | All of “virtual care” |
| 14 | 7 or 8 or 9 | 3630143 | All of “older adult” |
| 15 | 10 or 11 | 217462 | All of “Australia” |
| 16 | 12 and 13 and 14 and 15 | 2438 | Combine all hedges |
| 17 | limit 16 to (english and last 10 years) | 1820 | Apply general limiters |

**Supplementary Appendix 2 – Inclusion and exclusion criteria**

| **Parameter** | **Inclusion criteria** | **Exclusion criteria** |
| --- | --- | --- |
| **Population** | Studies which included older adults (defined as people aged >65 years)  Studies which had a participant mean/median age of approximately >=65 years  Studies **clearly stating** that it is addressing the older adult population with other definition (e.g., age >55 years) | Studies which excluded older adults (people aged >65 years)  Studies which included older adults and younger population but did not clearly stratify results for older adults (hence 'diluting' the results)  Studies addressing **only** caregiver of older adults  Studies addressing **only** healthcare professionals or students of medical and health professions |
| **Intervention** | Any initiatives underpinned by information technology and used for remote delivery of care between patients and professional healthcare provider  Modalities may include (but are not limited to): videoconference, apps, phone, SMS, databases, emails, websites, monitoring devices, etc.  Interactions between patient and healthcare provider may be synchronous or asynchronous | Non virtual care (e.g., face-to-face care)  Interventions using DVDs and radios  Using telecommunication means for data collection for a non-virtual care study (e.g., phone-based or email-based surveys)  Initiatives to improve knowledge or skills of healthcare providers or medical, nursing, or allied health students  Initiatives without healthcare provider supervision, such as those predominantly described as:   - Health promotion via booklets, Youtube channels, etc. - Self-assessment (e.g., self-supervision for risk of a certain disease using an online tool)   Outcomes without clear component of “healthcare delivery” |
| **Comparator** | Any suitable comparator or no comparator | N/A |
| **Outcomes** | The initiative had a clear objective in addressing certain care purpose for older adults, and/or health or behavioral outcomes in older adults, under the supervision of healthcare professionals.   - Care purpose broadly include the following: curative, preventive, rehabiliatitive, care access and long-term care improvement. - Health outcomes include (but are not limited to) the following: quality of life, hospitalisation, mortality, specific disease scoring system e.g., depressive score, etc. - Behavioural outcomes include (but are not limited to) the following: dietary choices, exercise frequency, attitude towards virtual care, etc.   The outcomes of interest outlined in the study should be provided from the perspective of older adults or in context of older adult care.  Studies reporting economic outcomes or cost-effectiveness analysis will be included, and the original study (e.g., randomised controlled trial or implementation study) associated with the economic evaluation will be sourced and included, if any. | No clear care purpose or health or behavioural outcomes provided as per description in inclusion criteria. |
| **Study type** | Quantitative studies (randomised or nonrandomised trials, cohort, case control, pre-and-post designs), qualitative evaluation studies and government evaluation reports | Reviews, case reports, commentaries, conference proceedings, thesis dissertations, (only references were screened)  Protocols without results and ongoing initiatives were collected and presented to VCHRI advisory group, however these have been excluded in this manuscript  Evaluation of virtual care in a broad/more general sense (i.e., not a single, purpose-driven initiative) |
| **Setting** | Population: older adults residing in general community, residential aged care facilities or receiving care in-hospital/clinic in Australia  Intervention: run by RACFs, hospitals, clinics or other relevant health or government organisations in Australia | Population: older adults not residing in Australia  Intervention: not run in the Australian healthcare setting, and not run by healthcare professional (e.g. peer-support where peer is defined as family, friends or non-health professional champions) |
| **Country** | Australia only  If multi-country, only if Australia-specific is provided | Non-Australia  If multi-country and Australia-specific results are **not** provided |
| **Language** | Studies in English | Studies NOT in English |
| **Year** | Studies from the last 10 years (2011-2021) | Studies NOT from the last 10 years |

**Supplementary Appendix 3 – PRISMA flowchart**

**Identification of studies via other methods**

**Identification of studies via databases**

Records identified from:

Medline (n = 1833)

Embase (n = 2374)

PsyInfo (n = 218)

CINAHL Plus (n = 1396)

AgeLine (n = 118)

Informit (n = 57)

INAHTA (n = 164)

**TOTAL (n = 6160)**

Records identified from:

Google Advanced Search, including APO (n = 121)

Handsearching (n = 5)

Citation searching (n = 10)

**TOTAL (n = 136)**

Records removed *before screening:*

Duplicate records removed

(n = 2501)

Records excluded based on title screening

(n = 84)

**Identification**

Records excluded, *irrelevant to review scope*

(n = 3168)

Records screened

(n = 3659)

Records excluded due to irrelevant content

(n = 12)

Records assessed for potential screening of full reports

(n = 52)

Reports not retrieved

(n = 0)

Reports sought for retrieval

(n = 491)

**Screening**

Reports excluded:

Wrong population (n = 153)

Wrong intervention (n = 91)

Wrong study type (n = 119)

Non-Australia (n = 27)

Others (n = 13)

Reports excluded:

Not evaluation of a targeted virtual care initiative (n = 25)

Others (n=9)

Full-text reports screened

(n = 40)

Reports assessed for eligibility

(n = 491)

Studies from databases (n = 88)

Additional studies/reports (n=6)

**TOTAL (n = 94) wherein 80 were unique initiatives**

**Included**

**Supplementary Appendix 4 – Definitions of WHO Digital Health Framework Items**

Taken from original published document.^1^

| **No.** | **Criterion** | **Description** |
| --- | --- | --- |
| **1** | Infrastructure | Presents the availability of infrastructure to support technology operations in the study location. This refers to physical infrastructure such as electricity, access to power and connectivity in the local context. |
| **2** | Technology platform | Describes and provides justification for the technology architecture. This includes a description of software and hardware and details of any modifications made to publicly available software. |
| **3** | Interoperability | Describes how the digital health strategy can integrate into existing health information system. Refers to whether the potential of technical and structural integration into existing HIS or programme has been described irrespective of whether such integration has been achieved by the existing system. |
| **4** | Intervention delivery | Describes the delivery of the digital health intervention. This should include frequency of mobile communication, mode of delivery of intervention (i.e. SMS, face-to-face, interactive voice response), timing and duration over which delivery occurred. |
| **5** | Intervention content | Describes details of the content of the intervention. Source and any modifications of the intervention content is described. |
| **6** | Usability/content testing | Describes formative research and/or content and/or usability testing with target group(s) clearly identified, as appropriate. |
| **7** | User feedback | Describes user feedback about the intervention or user satisfaction with the intervention. User feedback could include user opinions about content or user interface, their perceptions about usability, access, connectivity. |
| **8** | Access of individual participants | Mentions barriers or facilitators to the adoption of the intervention among study participants. Relates to individual-level structural, economic and social barriers or facilitators to access such as affordability, and other factors that may limit a user’s ability to adopt the intervention. |
| **9** | Cost assessment | Presents basic costs assessment of the digital health intervention from various perspectives. This criterion broadly refers to the reporting of some cost considerations for the digital health intervention in lieu of a full economic analysis. If a formal economic evaluation has been undertaken, it should be mentioned with appropriate references. |
| **10** | Adoption inputs/programme entry | Describes how people are informed about the programme including training, if relevant. Includes description of promotional activities and/or training required to implement the digital health intervention among the user population of interest. |
| **11** | Limitations for delivery at scale | Clearly presents the digital health intervention’s limitations for delivery at scale. |
| **12** | Contextual adaptability | Describes the adaptation, or not, of the solution to a different language, different population or context. Any tailoring or modification of the intervention that resulted from pilot testing/usability assessment is described. |
| **13** | Replicability | Details the intervention to support replicability. Clearly presents the source code/screenshots/flowcharts of the algorithms or examples of messages to support replicability of the digital health intervention in another setting. |
| **14** | Data security | Describes the data security procedures/confidentiality protocols. |
| **15** | Compliance with national guidelines or regulatory statutes | Details the mechanism used to assure that content or other guidance/information provided by the intervention is in alignment with existing national/regulatory guidelines is described. |
| **16** | Fidelity of the intervention | Describes the strategies employed to assess the fidelity of the intervention (i.e. was the intervention delivered as planned?). This may include assessment of participant engagement, use of back-end data to track message delivery and other technological challenges in the delivery of the intervention. |

**Supplementary Appendix 5 – Additional data synthesis**

**Intervention delivery (continued)**

Duration of virtual care varied depending on complexity of content and/or delivery mode. Videoconferences lasted up to 1 hour for new patients or major review sessions^2-4^ and between 5-15 minutes for follow-ups.^4-6^ Phone-based initiatives were often spread over multiple (6-24) sessions^7-11^ and lasted 10-30 minutes for supportive care^7, 8, 12-16^ and up to 1 hour for in-depth coaching sessions.^17, 18^ Two telemonitoring studies reported patients spent 1-5 minutes entering data.^19, 20^ Web-based interventions lasted between 3-18 months.^21, 22^

**Infrastructure & technology platform**

Initiatives employing apps (n=4)^23-27^ and web-based technologies (n=10)^21, 22, 28-38^ were often supplemented by other semi-automated systems, such as databases,^14, 37^ personalised short message service^14, 39^ and emails.^14, 40^ The majority of initiatives employing devices were monitoring systems which were available with peripherals capable of collecting and transmitting multiple physiological measures (n=9).^20, 41-50^ Devices used included iPad (n=2),^51, 52^ pedometer (n=3),^11, 40, 53^ and a dance training device.^54-57^

**Adaptability**

Fifteen studies indicated adaptability elements,^6, 21, 47, 48^ identifying or adapting to more health conditions or demographic profile,^12, 16, 26, 35, 39, 41, 44, 45, 58^ catering to patient preference, ^42^ improving workflow^59^ and following the latest available guidelines.^14, 16, 17, 27^

**Interoperability/scalability**

In general, no issues regarding interoperability were identified. Seventeen initiatives reported integrating virtual care onto existing infrastructure/systems.^3, 4, 30-32, 35, 41, 50, 53, 60-72^ Several studies developed novel infrastructure, which include new interoperable and intricate software and hardware systems (n=7),^14, 19, 37, 39, 44, 45, 58, 73, 74^ apps (n=3),^23, 25, 26^ device (n=3)^20, 51, 59^ and web-based modalities (n=11).^19-21, 33, 34, 46-49, 52, 54-57, 71^ A number of initiatives helped establish infrastructure for patients, for example by lending devices (n=7)^12, 26, 44, 45, 58, 69, 75-77^ or internet connectivity (n=4).^27, 69, 73, 78, 79^ Some initiatives were software-, app- or device-agnostic (n=4),^2, 6, 69, 80^ including the use of patient’s own device (n=2).^12, 81^

Information regarding scalability of initiatives was scarce, however there were reports of scale-up plans,^76^ established expansion,^65, 72, 82^ adoption,^31, 32^ implementation rate,^54-57, 83^ and clear implications of the pilot study.^42, 49, 80^

**Replicability**

In terms of replicability considerations, all initiatives provided sufficient details of the virtual care intervention or component involved, and 75% provided infrastructure details. Several studies also reported decision or clinical pathways incorporated into the virtual care design.^15, 26^ Provision of blueprint or ‘screenshots’ of the software interface^26, 27, 29, 69, 71^ as well as the hardware (device)^54-57, 68, 72, 84^ further enhanced replicability.

**Data security**

Twenty-three initiatives explicitly indicated data security compliance,^16, 19, 20, 32, 33, 37, 41-45, 50, 52, 58, 60, 61, 75, 81, 85-87^ with one reporting potential lack of security robustness.^51^

**Compliance with national guidelines or regulatory statutes**

Most initiatives demonstrated alignment with regulatory statues through funding source, including from federal (n=52) ^3, 6, 7, 10, 13-15, 17, 19-23, 29, 30, 33, 36-38, 40, 41, 46, 48-53, 57, 58, 61, 64, 69, 72, 74, 76, 82, 84, 86, 88-91^ and state government agencies (n=16) ^3, 10, 39, 52, 64, 67, 72, 75, 77, 78, 82, 85, 92-97^ philanthropic (n=7),^9, 13, 18, 22, 61, 72, 82, 98^ commercial organisations (n=5),^13, 16, 61, 86, 99^ hospital (n=5),^3, 7, 10, 64, 78, 100^ and university (n=8).^7, 10, 12, 26, 27, 35, 94, 101^ Funding was also in the form of direct provision of equipment and clinical service.^3, 63, 64^ Other examples of compliance included statement of alignment of virtual care (content or mode of delivery) with national policy for health outcomes,^2, 10, 16^ virtual care policy,^72, 82, 86^ general subsidy policy for individualised care for older adults^70^ or government-released initiatives.^25, 34^

**Large versus small studies (in terms of sample size)**

Initiatives which measured effectiveness (n=37) were all based on results of RCTs or implementation trials. Our summary discussion was drawn from all the initiatives, which included 22 individual relevant studies (RCTs or implementation trials) each with total sample size of >100. The rest of the studies (n=15) had a sample size of <100, and one of these studies had n<50.

Of all the initiatives included in this review, there were 27 (out of 80 initiatives, 34%) with a total sample size of <100. There were 4 studies with n<20, which were all feasibility studies and qualitative and/or mixed methods in nature. The findings of the studies were all related to older people’s feedback regarding virtual care and summarised in the review.

**References**

1. WHO. Monitoring and evaluating digital health interventions. A practical guideline to conducting research and assessment. 2016. Geneva, Switzerland: World Health Organisation.

2. Tam A, Leung A, O'Callaghan C and Fagermo N. Role of telehealth in perioperative medicine for regional and rural patients in Queensland. Internal Medicine Journal. 2017; 47: 933-937. DOI: 10.1111/imj.13484.

3. Burns CL, Ward EC, Hill AJ, Malcolm K, et al. A pilot trial of a speech pathology telehealth service for head and neck cancer patients. Journal of Telemedicine & Telecare. 2012; 18: 443-446. DOI: 10.1258/jtt.2012.GTH104.

4. Sabesan S, Larkins S, Evans R, Varma S, et al. Telemedicine for rural cancer care in North Queensland: bringing cancer care home. The Australian journal of rural health. 2012; 20: 259-264.

5. Moyle W, Jones C, Murfield J and Liu F. 'For me at 90, it's going to be difficult': feasibility of using iPad video-conferencing with older adults in long-term aged care. Aging & mental health. 2020; 24: 349-352. DOI: <http://dx.doi.org/10.1080/13607863.2018.1525605>.

6. Jones N. Final report of the Better Health Care Connections Video Consultation Project in the Frankston and Mornington Peninsula region. 2017. South Eastern Melbourne Primary Health Network.

7. Walters J, Cameron-Tucker H, Wills K, Schuz N, et al. Effects of telephone health mentoring in community-recruited chronic obstructive pulmonary disease on self-management capacity, quality of life and psychological morbidity: A randomised controlled trial. BMJ Open. 2013; 3: e003097. DOI: <http://dx.doi.org/10.1136/bmjopen-2013-003097>.

8. Walters JA, Cameron-Tucker H, Courtney-Pratt H, Nelson M, et al. Supporting health behaviour change in chronic obstructive pulmonary disease with telephone health-mentoring: insights from a qualitative study. BMC family practice. 2012; 13: 55. DOI: <http://dx.doi.org/10.1186/1471-2296-13-55>.

9. Lahham A, McDonald CF, Moore R, Cox NS, et al. The impact of home-based pulmonary rehabilitation on people with mild chronic obstructive pulmonary disease: A randomised controlled trial. Clinical Respiratory Journal. 2020; 14: 335-344. DOI: <http://dx.doi.org/10.1111/crj.13138>.

10. Cameron-Tucker HL, Wood-Baker R, Joseph L, Walters JA, et al. A randomized controlled trial of telephone-mentoring with home-based walking preceding rehabilitation in COPD. International journal of chronic obstructive pulmonary disease. 2016; 11: 1991-2000. DOI: <https://dx.doi.org/10.2147/COPD.S109820>.

11. Haynes A, Sherrington C, Wallbank G, Lester D, et al. "Someone's Got My Back": Older People's Experience of the Coaching for Healthy Ageing Program for Promoting Physical Activity and Preventing Falls. J Aging Phys Act. 2020; 29: 296-307. 2020/09/11. DOI: 10.1123/japa.2020-0116.

12. Brickwood KJ, Ahuja KDK, Watson G, O'Brien JA, et al. Effects of Activity Tracker Use With Health Professional Support or Telephone Counseling on Maintenance of Physical Activity and Health Outcomes in Older Adults: Randomized Controlled Trial. JMIR mHealth and uHealth. 2021; 9: e18686. DOI: <http://dx.doi.org/10.2196/18686>.

13. Williams A, Manias E, Walker R and Gorelik A. A multifactorial intervention to improve blood pressure control in co-existing diabetes and kidney disease: a feasibility randomized controlled trial. Journal of Advanced Nursing (John Wiley & Sons, Inc). 2012; 68: 2515-2525. DOI: 10.1111/j.1365-2648.2012.05950.x.

14. Cadilhac DA, Andrew NE, Busingye D, Cameron J, et al. Pilot randomised clinical trial of an eHealth, self-management support intervention (iVERVE) for stroke: feasibility assessment in survivors 12-24 months post-event. Pilot and Feasibility Studies. 2020; 6: 172. DOI: <http://dx.doi.org/10.1186/s40814-020-00706-x>.

15. Tutty E, Petelin L, McKinley J, Young M-A, et al. Evaluation of telephone genetic counselling to facilitate germline BRCA1/2 testing in women with high-grade serous ovarian cancer. European Journal of Human Genetics. 2019; 27: 1186-1196. DOI: <https://dx.doi.org/10.1038/s41431-019-0390-9>.

16. Hammersley ML, Cann VR, Parrish AM, Jones RA, et al. Evaluation of the effects of a telephone-delivered health behaviour change program on weight and physical activity. Nutrition & Dietetics. 2015; 72: 356-362. DOI: 10.1111/1747-0080.12213.

17. Tang D, Mitchell P, Liew G, Burlutsky G, et al. Telephone-Delivered Dietary Intervention in Patients with Age-Related Macular Degeneration: 3-Month Post-Intervention Findings of a Randomised Controlled Trial. Nutrients. 2020; 12: 3083. DOI: 10.3390/nu12103083.

18. Almeida OP, Patel H, Kelly R, Ford A, et al. Preventing depression among older people living in rural areas: A randomised controlled trial of behavioural activation in collaborative care. International journal of geriatric psychiatry. 2021; 36: 530-539. DOI: <https://dx.doi.org/10.1002/gps.5449>.

19. Elliot Bereznicki LR, Jackson SL and Peterson GM. Supervised patient self-testing of warfarin therapy using an online system. Journal of Medical Internet Research. 2013; 15: e138-131. DOI: 10.2196/jmir.2255.

20. De San Miguel K, Smith J and Lewin G. Telehealth remote monitoring for community-dwelling older adults with chronic obstructive pulmonary disease. Telemedicine Journal and e-Health. 2013; 19: 652-657. DOI: <http://dx.doi.org/10.1089/tmj.2012.0244>.

21. Alley SJ, Kolt GS, Duncan MJ, Caperchione CM, et al. The effectiveness of a web 2.0 physical activity intervention in older adults - a randomised controlled trial. International Journal of Behavioral Nutrition and Physical Activity. 2018; 15: 4. DOI: <http://dx.doi.org/10.1186/s12966-017-0641-5>.

22. Burns P, Jones SC, Iverson D and Caputi P. AsthmaWise-a field of dreams? the results of an online education program targeting older adults with asthma. Journal of Asthma. 2013; 50: 737-744. DOI: <http://dx.doi.org/10.3109/02770903.2013.799688>.

23. Bhattarai P, Newton-John TRO and Phillips JL. Apps for pain self-management of older people's arthritic pain, one size doesn't fit all: A qualitative study. Archives of Gerontology & Geriatrics. 2020; 89: 104062. DOI: 10.1016/j.archger.2020.104062.

24. Bhattarai P, Newton-John TRO and Phillips JL. Feasibility evaluation of a pain selfmanagement app-based intervention among older people living with arthritic pain: Study protocol. Pilot and Feasibility Studies. 2019; 5: 57. DOI: <http://dx.doi.org/10.1186/s40814-019-0442-5>.

25. Thomas R, Michaleff ZA, Greenwood H, Abukmail E, et al. Concerns and Misconceptions About the Australian Government's COVIDSafe App: Cross-Sectional Survey Study. JMIR public health and surveillance. 2020; 6: e23081. DOI: <https://dx.doi.org/10.2196/23081>.

26. Tongpeth J, Du H, Barry T and Clark RA. Effectiveness of an Avatar application for teaching heart attack recognition and response: A pragmatic randomized control trial. Journal of Advanced Nursing (John Wiley & Sons, Inc). 2020; 76: 297-311. DOI: 10.1111/jan.14210.

27. Wonggom P, Nolan P, Clark RA, Barry T, et al. Effectiveness of an avatar educational application for improving heart failure patients' knowledge and self‐care behaviors: A pragmatic randomized controlled trial. Journal of Advanced Nursing (John Wiley & Sons, Inc). 2020; 76: 2401-2415. DOI: 10.1111/jan.14414.

28. Alley S, Van Uffelen JGZ, Schoeppe S, Parkinson L, et al. Efficacy of a computer-tailored web-based physical activity intervention using Fitbits for older adults: a randomised controlled trial protocol. BMJ Open. 2019; 9: e033305. DOI: <http://dx.doi.org/10.1136/bmjopen-2019-033305>.

29. Kiropoulos LA, Griffiths KM and Blashki G. Effects of a multilingual information website intervention on the levels of depression literacy and depression-related stigma in greek-born and italian-born immigrants living in australia: a randomized controlled trial. Journal of Medical Internet Research. 2011; 13: e34-e34. DOI: 10.2196/jmir.1527.

30. O'Moore KA, Newby JM, Andrews G, Hunter DJ, et al. Internet Cognitive-Behavioral Therapy for Depression in Older Adults With Knee Osteoarthritis: A Randomized Controlled Trial. Arthritis care & research. 2018; 70: 61-70. DOI: <https://dx.doi.org/10.1002/acr.23257>.

31. Staples LG, Fogliati VJ, Dear BF, Nielssen O, et al. Internet-delivered treatment for older adults with anxiety and depression: implementation of the Wellbeing Plus Course in routine clinical care and comparison with research trial outcomes. BJPsych open. 2016; 2: 307-313.

32. Titov N, Fogliati VJ, Staples LG, Gandy M, et al. Treating anxiety and depression in older adults: randomised controlled trial comparing guided v. self-guided internet-delivered cognitive-behavioural therapy. BJPsych open. 2016; 2: 50-58. DOI: 10.1192/bjpo.bp.115.002139.

33. Titov N, Dear BF, Ali S, Zou JB, et al. Clinical and Cost-Effectiveness of Therapist-Guided Internet-Delivered Cognitive Behavior Therapy for Older Adults With Symptoms of Depression: A Randomized Controlled Trial. Behavior Therapy. 2015; 46: 193-205. DOI: <http://dx.doi.org/10.1016/j.beth.2014.09.008>.

34. Torrens E and Walker SM. Demographic characteristics of Australian health consumers who were early registrants for opt-in personally controlled electronic health records. Health Information Management Journal. 2017; 46: 127-133. DOI: 10.1177/1833358317699341.

35. Vandelanotte C, Ammann R, De Vries H and Mummery K. Can a website-delivered computer-tailored physical activity intervention be acceptable, usable, and effective for older people? Journal of Science and Medicine in Sport. 2012; 15: S46-S47. DOI: <http://dx.doi.org/10.1016/j.jsams.2012.11.113>.

36. Wilson CJ, Flight IH, Turnbull D, Gregory T, et al. A randomised controlled trial of personalised decision support delivered via the internet for bowel cancer screening with a faecal occult blood test: the effects of tailoring of messages according to social cognitive variables on participation. BMC medical informatics and decision making. 2015; 15: 25. DOI: <http://dx.doi.org/10.1186/s12911-015-0147-5>.

37. Staffieri SE, Ruddle JB, Kearns LS, Barbour JM, et al. Telemedicine model to prevent blindness from familial glaucoma. Clinical & experimental ophthalmology. 2011; 39: 760-765. DOI: <https://dx.doi.org/10.1111/j.1442-9071.2011.02556.x>.

38. Menant JC, Migliaccio AA, Sturnieks DL, Hicks C, et al. Reducing the burden of dizziness in middle-aged and older people: A multifactorial, tailored, single-blind randomized controlled trial. PLoS Medicine. 2018; 15: 1-21. DOI: 10.1371/journal.pmed.1002620.

39. Regan AK, Bloomfield L, Peters I and Effler PV. Randomized Controlled Trial of Text Message Reminders for Increasing Influenza Vaccination. Annals of Family Medicine. 2017; 15: 507-514. DOI: 10.1370/afm.2120.

40. Pasalich M, Lee AH, Jancey J, Burke L, et al. Sustainability of a physical activity and nutrition program for seniors. Journal of Nutrition, Health & Aging. 2013; 17: 486-491. DOI: <https://dx.doi.org/10.1007/s12603-012-0433-1>.

41. Halcomb E, Purcell R, Hickman L and Smyth E. Telemonitoring is acceptable amongst community dwelling older Australians with chronic conditions. Collegian. 2016; 23: 383-390. DOI: 10.1016/j.colegn.2015.09.007.

42. Chow JSF, Gonzalez-Arce V, Knight A and Kohler F. Retrospective analysis of telemonitoring in Wollondilly, Australia. Journal of Integrated Care. 2018; 26: 150-157. DOI: 10.1108/JICA-10-2017-0032.

43. Church A. Virtual Clinical Care Home Telemonitoring Service: A hospital avoidance strategy in regional South Australia. 2019. SA Health.

44. My Health Clinic At Home pilot summary report. 2014. Australia: Feros Care & Southern Cross University.

45. My Health Clinic At Home Pilot final report. 2014. Australia: Feros Care & Southern Cross University.

46. Wade R, Shaw K and Cartwright C. Factors affecting provision of successful monitoring in home Telehealth. Gerontology. 2012; 58: 371-377. DOI: <https://dx.doi.org/10.1159/000335033>.

47. Ward EC, Sharma S, Burns C, Theodoros D, et al. Validity of conducting clinical dysphagia assessments for patients with normal to mild cognitive impairment via telerehabilitation. Dysphagia. 2012; 27: 460-472. 2012/01/25. DOI: 10.1007/s00455-011-9390-9.

48. Ward EC, Burns CL, Theodoros DG and Russell TG. Impact of dysphagia severity on clinical decision making via telerehabilitation. Telemedicine Journal and e-Health 2014; 20: 296-303. DOI: <http://dx.doi.org/10.1089/tmj.2013.0198>.

49. Evaluation of the In-Home Telemonitoring for Veterans trial. 2017. Glynde, South Australia: Health Outcomes International.

50. Celler B, Varnfield M, Sparks R, Li J, et al. Home monitoring of chronic disease for aged care. 2016. Australia: Australian e-Health Research Centre, CSIRO.

51. Tieman JJ, Swetenham K, Morgan DD, To TH, et al. Using telehealth to support end of life care in the community: a feasibility study. BMC Palliative Care. 2016; 15: 1-7. DOI: 10.1186/s12904-016-0167-7.

52. Katalinic O, Young A and Doolan D. Case study: the Interact Home Telehealth Project. Journal of Telemedicine & Telecare. 2013; 19: 418-424. DOI: 10.1177/1357633X13506513.

53. Wootton SL, Hill K, Alison JA, Ng LWC, et al. Effects of Ongoing Feedback During a 12-Month Maintenance Walking Program on Daily Physical Activity in People with COPD. Lung. 2019; 197: 315-319. DOI: <http://dx.doi.org/10.1007/s00408-019-00216-5>.

54. Schoene D, Valenzuela T, Toson B, Delbaere K, et al. Interactive Cognitive-Motor Step Training Improves Cognitive Risk Factors of Falling in Older Adults - A Randomized Controlled Trial. PLoS ONE. 2015; 10: e0145161. 2015/12/18. DOI: 10.1371/journal.pone.0145161.

55. Schoene D, Smith ST, Davies TA, Delbaere K, et al. A Stroop Stepping Test (SST) using low-cost computer game technology discriminates between older fallers and non-fallers. Age Ageing. 2014; 43: 285-289. 2013/10/17. DOI: 10.1093/ageing/aft157.

56. Schoene D, Lord SR, Verhoef P and Smith ST. A novel video game--based device for measuring stepping performance and fall risk in older people. Arch Phys Med Rehabil. 2011; 92: 947-953. 2011/05/10. DOI: 10.1016/j.apmr.2011.01.012.

57. Schoene D, Lord SR, Delbaere K, Severino C, et al. A randomized controlled pilot study of home-based step training in older people using videogame technology. PLoS ONE. 2013; 8: e57734. 2013/03/09. DOI: 10.1371/journal.pone.0057734.

58. Nancarrow S, Banbury A and Buckley J. Evaluation of a National Broadband Network-enabled Telehealth trial for older people with chronic disease. Australian Health Review. 2016; 40: 641-648. DOI: <http://dx.doi.org/10.1071/AH15201>.

59. Rolls D, Khanna S, Lloyd N, Reeson A, et al. Before-after evaluation of patient length of stay in a rehabilitation context following implementation of an electronic patient journey board. International Journal of Medical Informatics. 2020; 134: N.PAG-N.PAG. DOI: 10.1016/j.ijmedinf.2019.104042.

60. Dham P, Gupta N, Alexander J, Black W, et al. Community based telepsychiatry service for older adults residing in a rural and remote region- utilization pattern and satisfaction among stakeholders. BMC Psychiatry. 2018; 18: 316. DOI: <http://dx.doi.org/10.1186/s12888-018-1896-3>.

61. Venuthurupalli SK, Rolfe A, Fanning J, Cameron A, et al. Chronic Kidney Disease, Queensland (CKD.QLD) Registry: Management of CKD With Telenephrology. Kidney International Reports. 2018; 3: 1336-1343. DOI: <http://dx.doi.org/10.1016/j.ekir.2018.07.013>.

62. Wundersitz C, Caelli A, Georgy J, Musovic A, et al. Conducting community rehabilitation review sessions via videoconference: A feasibility study. Australian Journal of Rural Health. 2020; 28: 603-612. DOI: 10.1111/ajr.12665.

63. Burns CL, Kularatna S, Ward EC, Hill AJ, et al. Cost analysis of a speech pathology synchronous telepractice service for patients with head and neck cancer. Head and Neck. 2017; 39: 2470-2480. DOI: <http://dx.doi.org/10.1002/hed.24916>.

64. Burns CL, Ward EC, Hill AJ, Kularatna S, et al. Randomized controlled trial of a multisite speech pathology telepractice service providing swallowing and communication intervention to patients with head and neck cancer: Evaluation of service outcomes. Head and Neck. 2017; 39: 932-939. DOI: <https://dx.doi.org/10.1002/hed.24706>.

65. Lillicrap T, Pinheiro A, Miteff F, Garcia-Bermejo P, et al. No Evidence of the "Weekend Effect" in the Northern New South Wales Telestroke Network. Frontiers in Neurology. 2020; 11: 130. DOI: <http://dx.doi.org/10.3389/fneur.2020.00130>.

66. Marino R, Tonmukayakul U, Manton D, Stranieri A, et al. Cost-analysis of teledentistry in residential aged care facilities. Journal of Telemedicine & Telecare. 2016; 22: 326-332. DOI: 10.1177/1357633X15608991.

67. Mariño R, Tonmukayakul U, Marwaha P, Collmann R, et al. Teleconsultation/Telediagnosis using Teledentistry Technology: a Pilot Feasibility Study. 2014; 6.

68. Nagao KJ, Koschel A, Haines HM, Bolitho LE, et al. Rural Victorian Telestroke project. Internal Medicine Journal. 2012; 42: 1088-1095. DOI: <http://dx.doi.org/10.1111/j.1445-5994.2011.02603.x>.

69. Taylor A, Morris G, Pech J, Rechter S, et al. Home Telehealth Video Conferencing: Perceptions and Performance. JMIR mHealth and uHealth. 2015; 3: e90. DOI: <https://dx.doi.org/10.2196/mhealth.4666>.

70. Yu P, Hailey D, Fleming R and Traynor V. An exploration of the effects of introducing a telemonitoring system for continence assessment in a nursing home. Journal of Clinical Nursing (John Wiley & Sons, Inc). 2014; 23: 3069-3076. DOI: 10.1111/jocn.12538.

71. Karunanithi M and Zhang Q. An Innovative Technology to Support Independent Living: The Smarter Safer Homes Platform. Studies in health technology and informatics. 2018; 246: 102-110.

72. Bladin CF, Molocijz N, Ermel S, Bagot KL, et al. Victorian Stroke Telemedicine Project: implementation of a new model of translational stroke care for Australia. Internal Medicine Journal. 2015; 45: 951-956. DOI: 10.1111/imj.12822.

73. Theodoros DG, Hill AJ and Russell TG. Clinical and Quality of Life Outcomes of Speech Treatment for Parkinson's Disease Delivered to the Home Via Telerehabilitation: A Noninferiority Randomized Controlled Trial. American Journal of Speech-Language Pathology. 2016; 25: 214-232. DOI: 10.1044/2015_AJSLP-15-0005.

74. Wade V, Whittaker F and Hamlyn J. An evaluation of the benefits and challenges of video consulting between general practitioners and residential aged care facilities. Journal of Telemedicine & Telecare. 2015; 21: 490-493. DOI: 10.1177/1357633X15611771.

75. Jiang B, Bills M and Poon P. Integrated telehealth-assisted home-based specialist palliative care in rural Australia: A feasibility study. Journal of telemedicine and telecare. 2020: 1357633X20966466. DOI: <http://dx.doi.org/10.1177/1357633X20966466>.

76. Banbury A, Chamberlain D, Nancarrow S, Dart J, et al. Can videoconferencing affect older people's engagement and perception of their social support in long-term conditions management: a social network analysis from the Telehealth Literacy Project. Health & Social Care in the Community. 2017; 25: 938-950. DOI: 10.1111/hsc.12382.

77. Ding H, Jayasena R, Chen SH, Maiorana A, et al. The Effects of Telemonitoring on Patient Compliance With Self-Management Recommendations and Outcomes of the Innovative Telemonitoring Enhanced Care Program for Chronic Heart Failure: Randomized Controlled Trial. Journal of Medical Internet Research. 2020; 22: e17559. DOI: 10.2196/17559.

78. Hwang R, Bruning J, Morris NR, Mandrusiak A, et al. Home-based telerehabilitation is not inferior to a centre-based program in patients with chronic heart failure: a randomised trial. Journal of Physiotherapy (Elsevier). 2017; 63: 101-107. DOI: 10.1016/j.jphys.2017.02.017.

79. Hwang R, Morris NR, Mandrusiak A, Bruning J, et al. Cost-Utility Analysis of Home-Based Telerehabilitation Compared With Centre-Based Rehabilitation in Patients With Heart Failure. Heart, Lung & Circulation. 2019; 28: 1795-1803. DOI: 10.1016/j.hlc.2018.11.010.

80. Dorsey K. GP video consultations a success in residential aged care, <https://www.ruralhealth.org.au/partyline/article/gp-video-consultations-success-residential-aged-care> (2017, accessed 15 Apr 2021).

81. Stillerova T, Liddle J, Gustafsson L, Lamont R, et al. Could everyday technology improve access to assessments? A pilot study on the feasibility of screening cognition in people with Parkinson's disease using the Montreal Cognitive Assessment via Internet videoconferencing. Australian Occupational Therapy Journal. 2016; 63: 373-380. DOI: 10.1111/1440-1630.12288.

82. Bladin CF, Kim J, Bagot KL, Vu M, et al. Improving acute stroke care in regional hospitals: clinical evaluation of the Victorian Stroke Telemedicine program. Medical Journal of Australia. 2020; 212: 371-377. DOI: 10.5694/mja2.50570.

83. Ling R, Searles A, Hewitt J, Considine R, et al. Cost analysis of an integrated aged care program for residential aged care facilities. Australian Health Review. 2019; 43: 261-267. DOI: 10.1071/AH16297.

84. Mitchell BG, Northcote M, Cheng AC, Fasugba O, et al. Reducing urinary catheter use using an electronic reminder system in hospitalized patients: A randomized stepped-wedge trial. Infection Control & Hospital Epidemiology. 2019; 40: 427-431. DOI: 10.1017/ice.2019.31.

85. Beauchamp A, Mohebbi M, Cooper A, Pridmore V, et al. The impact of translated reminder letters and phone calls on mammography screening booking rates: Two randomised controlled trials. PLoS ONE. 2020; 15: e0226610. DOI: <http://dx.doi.org/10.1371/journal.pone.0226610>.

86. Lannin NA, Anderson C, Lim J, Paice K, et al. Telephone follow-up was more expensive but more efficient than postal in a national stroke registry. Journal of Clinical Epidemiology. 2013; 66: 896-902. DOI: <http://dx.doi.org/10.1016/j.jclinepi.2013.03.005>.

87. Sampurno F, Ruseckaite R, Millar JL and Evans SM. Comparison of Patient-Reported Quality-of-Life and Complications in Men With Prostate Cancer, Between Two Modes of Administration. Clinical Genitourinary Cancer. 2016; 14: 284-289. DOI: <http://dx.doi.org/10.1016/j.clgc.2015.12.016>.

88. White VM, Macvean ML, Grogan S, D'Este C, et al. Can a tailored telephone intervention delivered by volunteers reduce the supportive care needs, anxiety and depression of people with colorectal cancer? A randomised controlled trial. Psycho-Oncology. 2012; 21: 1053-1062. DOI: 10.1002/pon.2019.

89. Martin-Khan M, Flicker L, Wootton R, Loh PK, et al. The diagnostic accuracy of telegeriatrics for the diagnosis of dementia via video conferencing. J Am Med Dir Assoc. 2012; 13: 487.e419-424. 2012/05/11. DOI: 10.1016/j.jamda.2012.03.004.

90. Moayeri F, Dunt D, Hsueh YSA and Doyle C. Cost-utility analysis of telephone-based cognitive behavior therapy in chronic obstructive pulmonary disease (COPD) patients with anxiety and depression comorbidities: an application for willingness to accept concept. Expert Review of Pharmacoeconomics and Outcomes Research. 2019; 19: 331-340. DOI: <http://dx.doi.org/10.1080/14737167.2019.1536550>.

91. Provencher V, Clemson L, Wales K, Cameron ID, et al. Supporting at-risk older adults transitioning from hospital to home: who benefits from an evidence-based patient-centered discharge planning intervention? Post-hoc analysis from a randomized trial. BMC Geriatrics. 2020; 20: 84-84. DOI: 10.1186/s12877-020-1494-3.

92. Towers C and Tyler M. The broadband-enabled innovation program: a working demonstration of the effective use of technology in community-based patient care. Australian family physician. 2014; 43: 848-851.

93. Harrison JD, Young JM, Solomon MJ, Butow PN, et al. Randomized pilot evaluation of the supportive care intervention "CONNECT" for people following surgery for colorectal cancer. Diseases of the Colon and Rectum. 2011; 54: 622-631. DOI: <http://dx.doi.org/10.1007/DCR.0b013e31820bc152>.

94. Hullick CJ, Hall AE, Conway JF, Hewitt JM, et al. Reducing Hospital Transfers from Aged Care Facilities: A Large-Scale Stepped Wedge Evaluation. Journal of the American Geriatrics Society. 2021; 69: 201-209. DOI: <https://doi.org/10.1111/jgs.16890>.

95. Voukelatos A, Merom D, Sherrington C, Rissel C, et al. The impact of a home-based walking programme on falls in older people: the Easy Steps randomised controlled trial. Age and Ageing. 2015; 44: 377-383.

96. Young AM, Mudge AM, Banks MD, Rogers L, et al. Improving nutritional discharge planning and follow up in older medical inpatients: Hospital to Home Outreach for Malnourished Elders. Nutrition & Dietetics. 2018; 75: 283-290. DOI: 10.1111/1747-0080.12408.

97. Young JM, Butow PN, Walsh J, Durcinoska I, et al. Multicenter Randomized Trial of Centralized Nurse-Led Telephone-Based Care Coordination to Improve Outcomes After Surgical Resection for Colorectal Cancer: The CONNECT Intervention. Journal of Clinical Oncology. 2013; 31: 3585-3591. DOI: 10.1200/JCO.2012.48.1036.

98. Doyle C, Bhar S, Fearn M, Ames D, et al. The impact of telephone-delivered cognitive behaviour therapy and befriending on mood disorders in people with chronic obstructive pulmonary disease: A randomized controlled trial. Br J Health Psychol. 2017; 22: 542-556. 2017/05/26. DOI: 10.1111/bjhp.12245.

99. Gallagher C, Orchard J, Nyfort-Hansen K, Sanders P, et al. NursE led Atrial Fibrillation Management: The NEAT Study: A Randomized Controlled Trial. Journal of Cardiovascular Nursing. 2020; 35: 456-467. DOI: 10.1097/JCN.0000000000000680.

100. Padayachee A, Ranatunga C and Comans TA. Utilising capacity in a rural hospital to support older people requiring hospital care: Kilcoy Connect. Australian Journal of Rural Health. 2019; 27: 344-350. DOI: 10.1111/ajr.12475.

101. Jancey JM, Lee AH, Howat PA, Burke L, et al. The effectiveness of a walking booster program for seniors. American Journal of Health Promotion. 2011; 25: 363-367. DOI: 10.4278/ajhp.090512-ARB-164.
